# Genetic and pharmacologic inhibition of ALDH1A3 as a treatment of β-cell failure

**DOI:** 10.1038/s41467-023-36315-4

**Published:** 2023-02-02

**Authors:** Jinsook Son, Wen Du, Mark Esposito, Kaavian Shariati, Hongxu Ding, Yibin Kang, Domenico Accili

**Affiliations:** 1 Department of Medicine and Naomi Berrie Diabetes Center, Vagelos College of Physicians and Surgeons, Columbia University, New York, NY 10032 USA; 2 Kayothera Inc, Seattle, WA USA; 3 Department of Molecular Biology, Princeton University, 08544 Princeton, NJ USA; 4 Department of Pharmacy Practice & Science, College of Pharmacy, University of Arizona, Tucson, AZ 85721 USA

**Keywords:** Type 2 diabetes, Reprogramming, Mechanisms of disease, Differentiation

## Abstract

Type 2 diabetes (T2D) is associated with β-cell dedifferentiation. Aldehyde dehydrogenase 1 isoform A3 (ALHD1A3) is a marker of β-cell dedifferentiation and correlates with T2D progression. However, it is unknown whether ALDH1A3 activity contributes to β-cell failure, and whether the decrease of ALDH1A3-positive β-cells (A+) following pair-feeding of diabetic animals is due to β-cell restoration. To tackle these questions, we (i) investigated the fate of A+ cells during pair-feeding by lineage-tracing, (ii) somatically ablated ALDH1A3 in diabetic β-cells, and (iii) used a novel selective ALDH1A3 inhibitor to treat diabetes. Lineage tracing and functional characterization show that A+ cells can be reconverted to functional, mature β-cells. Genetic or pharmacological inhibition of ALDH1A3 in diabetic mice lowers glycemia and increases insulin secretion. Characterization of β-cells following ALDH1A3 inhibition shows reactivation of differentiation as well as regeneration pathways. We conclude that ALDH1A3 inhibition offers a therapeutic strategy against β-cell dysfunction in diabetes.

## Abstract (web summary)

β-cell dedifferentiation is a key feature of type 2 diabetes. Here, the authors show evidence of re-differentiation of de-differentiated β-cells and identify ALDH1A3 as a key player in this process, proposing inhibition of ALDH1A3 as a treatment method for β-cell dysfunction in diabetes.

## Introduction

Insulin secretion by pancreatic β-cells is a fundamental component of glucose homeostasis^1,2^. β-cells respond to metabolic demand by increasing insulin production and/or cell mass^3^. However, chronic metabolic stress^4^ leads to a decline in β-cell function and mass that contributes to the development of type 2 diabetes (T2D)^5^. β-cell dedifferentiation is a key mechanism underlying β-cell failure^6^ whereby β-cells lose their identity and dedifferentiate into non-functional endocrine progenitor-like cells^7–9^. In T2D patients, 4 weeks of a low-calorie diet can restore glucose control and chronic adoption of this diet can lead to lasting benefits on glycemia^10–15^. Similarly, when diabetic *db/db* mice are pair-fed to wildtype littermate levels, glycemia declines and insulin secretion increases^16^. These observations in humans and rodents are consistent with the possibility that β-cell function can be restored. Lineage-tracing experiments also support this idea^17–20^. In addition, CRISPR-mediated functional studies in human islets show that the T2D transcriptional signature can be reversed by targeted inhibition of a key master regulator of dedifferentiation, BACH2^21^.

ALDH1A3 (also known as RALDH3) is a marker of β-cell dedifferentiation in murine^22^ and human T2D^8,9^. A+ β-cells show reduced insulin secretion compared to ALDH1A3-negative (A–) β-cells^22^. Importantly, the number of A+ cells decreases significantly upon pair-feeding, suggesting that ALDH1A3 activity is dynamically correlated to β-cell function^16^. However, it is not known whether this is due to redifferentiation of A+ to functional, mature A– cells or death of A+ cells followed by β-cell generation. Most importantly, the strong inverse correlation between ALDH1A3 expression and β-cell function raises the question of whether ALDH1A3 activity drives β-cell dysfunction.

Here we generated two animal models to address these questions: (i) we engineered an ALDH1A3-Cre^ert^ knock-in allele for lineage-tracing of A+ cells; (ii) to investigate the role of ALDH1A3 in dedifferentiating β-cells, we somatically deleted it in this cell type. Lineage-tracing experiments demonstrate that A+ cells can convert back to mature, functional A– cells following pair-feeding. Furthermore, β-cell-specific knockout of *Aldh1a3* in *db/db* mice lowered glucose levels and increased insulin secretion in vivo and ex vivo. To support these conclusions and validate the translational relevance of these findings, we administered a novel selective ALDH1A3 inhibitor, KOTX1, to *db/db* or diet-induced diabetic mice (DIO) as well as human T2D islets, and show that it improved glucose control, increased insulin secretion, and enhanced glucose tolerance.

To understand the mechanism of β-cell restoration, we surveyed mRNA expression. Remarkably, we observed that Aldh1a3 KO β-cells activated pancreatic regeneration and β-cell proliferation pathways, including the Reg gene family, and increased β-cell proliferation in a retinoic acid (RA)-dependent fashion.

The findings that A+ β-cells can be converted to mature, functional β-cells by pair-feeding, and that genetic or pharmacologic inhibition of ALDH1A3 can improve β-cell dedifferentiation and dysfunction nominate ALDH1A3 as a potential therapeutic target in the treatment of T2D.

## Results

### Lineage-tracing ALDH1A3-positive β-cells

**Fig. 1:**
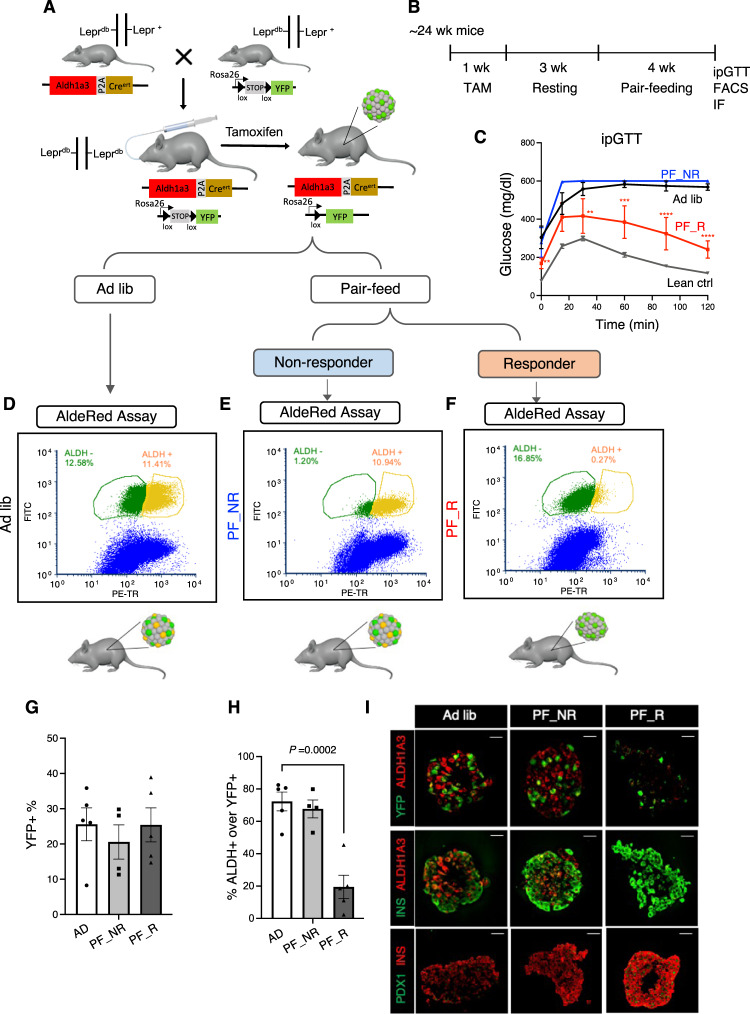
A+ β-cells are converted to A– β-cells. **A** Generation of *Aldh1a3-Cre*^*ert*^*:YFP*^*fl/+*^ *db/db* mice and tracing of A+ cells in *db/db* mice during pair-feeding. **B** Schematic drawing of the experimental procedure. **C** IPGTT after 4 weeks of pair-feeding (*n* = 6, 5, 4 or 10 for ad lib, PF_R (*Pair-fed responder)*, PF_NR (*Pair-fed non-responder)* or lean mice. Data are expressed as means ± SEM. Two-way ANOVA with multiple comparison test was used for statistical analysis. The *P* value was 0.0097, 0.0077, 0.0002 for time points 0, 30, or 60 min and *P* < 0.0001 for 90 and 120 min in comparison of ad lib with PF_R. *P < 0.05, **P < 0.01, ***P < 0.001, ****P < 0.0001. **D**–**F** FACS to detect lineage-traced A+ cells before Pair-feeding (YFP+) and cells with ongoing ALDH1A3 activity (Red+) in *Ad lib* (**D**), PF_NR (**E**) or PF_R (**F**). **G**, **H** Quantification of YFP+ (**G**) cells or YFP+/ongoing ALDH1A3+ cells (**H**). All data are expressed as means ± SEM for *n* = 5, 4, or 5 biologically independent samples per group. One-way ANOVA with multiple comparison test was used for statistical analysis. **I** Immunofluorescent staining for ALDH1A3, YFP, PDX1 and Insulin using isolated islets in each condition as in (**A**). Representative immunofluorescence images of *n* = 4 mice per group. Scale bars: 20 μM. Source data are provided as a Source Data file.

Expression of ALDH1A3 increases with β-cell dedifferentiation^22^, and decreases under pair-feeding in *db/db* mice^16^. However, these experiments do not demonstrate whether A+ cells revert to mature, functional A– cells, or die and are replaced by new β-cells. To answer this question, we generated *Aldh1a3-Cre*^*ert*^ mice harboring a tamoxifen-activable Cre (Cre^ert^) knocked into the *Aldh1A3* locus for lineage-tracing experiments (Fig. 1A). To introduce a fluorescent reporter gene to monitor Aldh1a3 expression, we crossed *Aldh1a3*^*+/CreERT*^ with *Rosa26-lox-STOP-lox-YFP (R26R-YFP)* mice to generate *Aldh1a3-Cre*^*ert*^:R26R-YFP double knock-in mice. Finally, we backcrossed the double knock-in onto a *db/db* background to generate *Aldh1a3*^*+/CreERT*^*:YFP*^*fl/+*^*:Lepr*^*db/db*^ mice and monitor the fate of A+ cells in response to pair-feeding. First, we assessed the efficiency of labeling A+ cells by administering tamoxifen to *Aldh1a3-Cre*^*ert*^ *db/db* mice for 5 days (Fig. 1B). We scored >20% of pancreatic islet cells as YFP+ (Supplementary Fig. 1A), demonstrating the efficiency of lineage-tracing. In separate cohorts of *Aldh1a3-Cre*^*ert*^ *db/db* and control mice, we administered tamoxifen then pair-fed them for 4 weeks (Fig. 1B–F). Consistent with previous findings, we observed lower fasting glucose and improved GTT in ~2/3 of pair-fed animals (*Pair-fed responder)* (Fig. 1C and Supplementary Fig. 1B) compared to ad libitum-fed (*ad lib*) *db/db* mice, whereas ~1/3 mice did not respond to pair-feeding (*pair-fed non-responder*)^16^.

To assess the conversion of A+ cells into A– cells following pair-feeding, we isolated islets from each group of pair-fed animals and incubated them with AldeRed, an ALDH-activatable red fluorescent substrate^23^ that labels cells possessing active ALDH1A3. This labeling strategy combined with the YFP reporter distinguishes A+ cells that have reverted to A– cells (YFP+/Red–) from A+ cells with ongoing ALDH1A3 activity (YFP+/Red+) (Fig. 1D–F). We set ALDH+ population gates using N,N Diethylaminobenzaldehyde, a broad ALDH enzymatic inhibitor (Supplementary Fig. 1C) and quantified each population by flow cytometry. The number of YFP+ cells was comparable (~25%) in pair-feeding responders, pair-feeding non-responders, and *ad lib* conditions (Fig. 1G). These data indicate that cell death cannot account for the decrease in A+ cells in pair-feeding responders. In contrast, AldeRed+ cells were significantly lower in responders compared to non-responders or *ad lib* mice (Fig. 1D–H), consistent with prior results^16^. Most interestingly, ~80% of YFP+ cells lacked detectable ALDH activity in responders (Fig. 1H), showing that A+ cells converted back to A– cells (Red–) as a result of pair-feeding (Fig. 1A–H). These data indicate that dedifferentiated β-cells can re-differentiate.

We tested this striking finding by immunofluorescence (Fig. 1I). Consistent with the FACS data, *Pair-fed responders* showed YFP+ cells without ALDH1A3 protein expression whereas *pair-fed non-responders* and *ad lib db/db* showed double-positive YFP+/ALDH1A3 cells (Fig. 1I). Furthermore, YFP+/A– cells showed increased insulin content, PDX1 expression (Fig. 1I), and insulin secretion (Supplementary Fig. 1D), supporting the conclusion that A+ cells can be converted back to functional, mature A– β-cells. Therefore, lineage tracing of A+ cells demonstrates that improvement of diabetes in *Pair-fed responder db/db* mice is associated with reversal of ALDH1A3 activation, whereas worsening β-cell failure is associated with an increase in A+ cells.

### Aldh1a3 KO in β-cells restores β-cell function

The increase in insulin content and secretion in A+ cells reconverted to A– cells after pair-feeding suggests that ALDH1A3 plays a role in β-cell dedifferentiation and/or dysfunction. ALDH1A3 ablation may therefore improve or reverse diabetes in mice. To test this hypothesis, we generated somatic ablation of *Aldh1a3* in β-cells *(RIP-Cre*^*herr*^*:Aldh1a3*^*fl/fl*^*: tdT*^*fl/+*^) using *RIP-Cre*^*herr*^ with a β-cell-restricted pattern of recombination^24^ (Supplementary Fig. 2A). We backcrossed this allele onto a *db/db* background to generate *β-*cell-specific *Aldh1a3 KO_db/db* mice *(RIP-Cre*^*Herr*^*:Aldh1a3* ^*fl/fl*^*:tdT*^*fl/+*^*:Lepr*^*db/db*^).

**Fig. 2:**
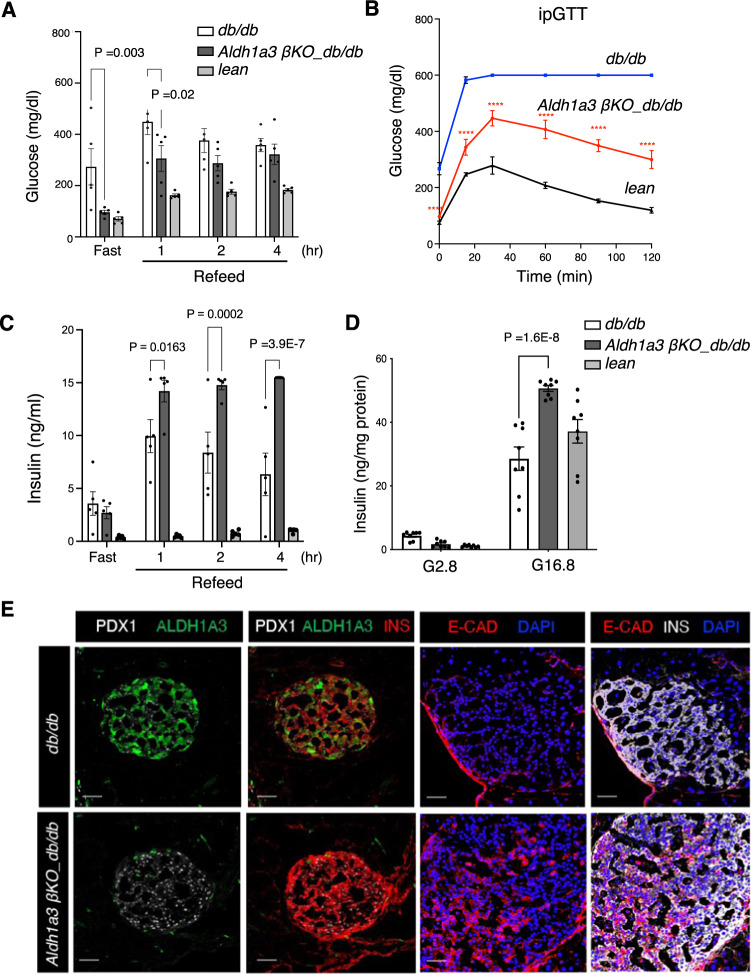
β-Aldh1a3 KO improves diabetic phenotypes. **A** Glucose levels in fasted and refed 12-week-old male mice (*n* = 5 mice per genotype). **B** IPGTT in 20-week-old mice (*n* = 5 per genotype). The *P* value was 1.6E–8, 1.0E–15, 2.6E–7, 3.3E–10, 1.0E–15, or 1.0E–15, for each time point in comparison of Aldh1a3 KO_*db/db* with *db/db* mice. *P < 0.05, **P < 0.01, ***P < 0.001, ****P < 0.0001. **C** Plasma insulin levels in fasted and refed 12-week-old male mice (*n* = 5 per genotype). **D** Insulin secretion in islets from β-Aldh1a3 KO_*db/db*, lean or *db/db* mice. ANOVA was performed between the two groups (*n* = 8 per group). **E** Immunofluorescent staining of PDX1, INS, E-Cadherin and ALDH1A3 in β-Aldh1a3 KO_*db/db* or *db/db* mice. Representative immunofluorescence images of *n* = 3 mice per group. Scale bars: 50 μM. All data are expressed as means ± SEM. Two-way ANOVA with multiple comparison test was used for statistical analysis for (**A**–**D**). Source data are provided as a Source Data file.

Immunohistochemistry and mRNA measurement in sorted β-cells confirmed ALDH1A3 ablation (Supplementary Fig. 2B, C). *β-Aldh1a3 KO_db/db* showed increased insulin expression compared to *db/db* controls (Supplementary Fig. 2B, C). To assess the effect of *β-Aldh1a3 KO_db/db* on glucose homeostasis, we measured fasting and refed glycemia. *β-Aldh1a3 KO_db/db* maintained fasting glucose levels comparable to *db/+* (*RIP-Cre*^*Herr*^*:Aldh1a3*^*fl/+*^*:tdT*^*fl/+*^*:Lepr*^*db/+*^*)* and had lower glucose levels than *db/db* mice (*RIP-Cre*^*Herr*^*:Aldh1a3*^*fl/+*^*:tdT*^*fl/+*^*:Lepr*^*db/db*^*)* upon refeeding (Fig. 2A). Furthermore, *β-Aldh1a3 KO_db/db* mice demonstrated significantly improved glucose tolerance during IPGTT (Fig. 2B). Plasma insulin levels were higher in *β-Aldh1a3 KO_db/db* compared to *db/db* mice (Fig. 2C), suggesting that improved β-cell function underlies the improved glucose control. Ex vivo glucose-stimulated insulin secretion (GSIS) assays confirmed this finding, as *β-cell Aldh1a3 KO_db/db* islets secreted 50% more insulin in response to 16.8 mM glucose than *db/db* or db/+ islets (Fig. 2D). Immunohistochemistry demonstrated increased PDX1, NKX6.1^22^, E-Cadherin^25,26^ and MAFA levels in *β-Aldh1a3 KO_db/db* islets (Fig. 2D and Supplementary Fig. 2D, E). These findings indicate that ablating ALDH1A3 in diabetic animals improves β-cell function and maturity, resulting in lower glycemia and higher insulin secretion.

### Pharmacological inhibition of ALDH1A3 improves diabetes

**Fig. 3:**
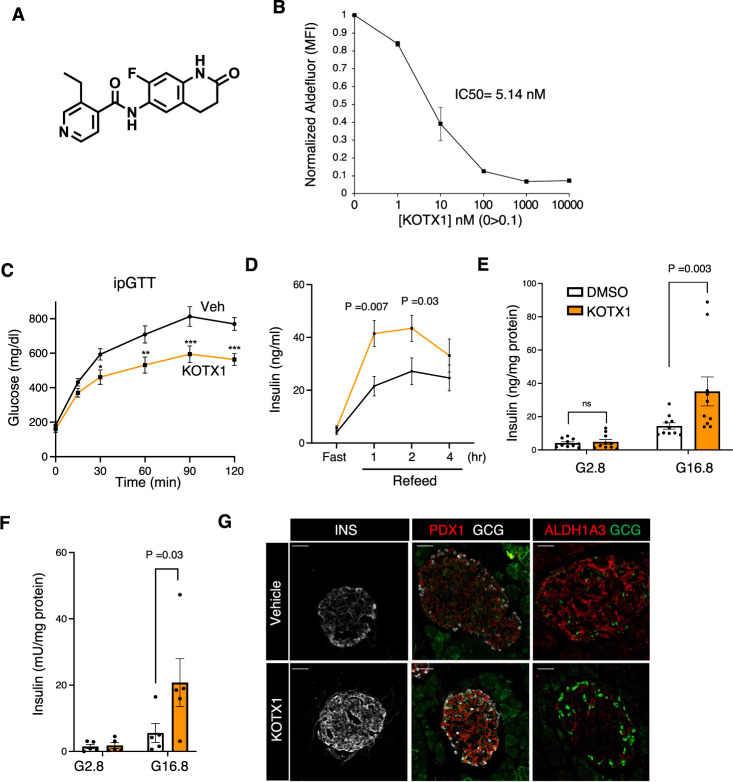
ALDH1A3 inhibitors improve β-cell function. **A** KOTX1 structure. **B** KOTX1 activity was measured by Aldefluor assay in A375 cells. **C** IPGTT after 4 weeks of treatment. The *P* value was 0.0154, 0.0012, 8.4E–5, or 0.0002, for 30, 60, 90 or 120 min in comparison of KOTX1 with vehicle. *P < 0.05, **P < 0.01, ***P < 0.001, ****P < 0.0001 (*n* = 12 mice per group). **D** Plasma insulin levels in *db/db* mice treated with KOTX1 after 16-h fasting or refeeding (*n* = 12 mice per group). **E** Insulin secretion in islets from *db/db* mice following 3 days in vitro treatment with 10 μM KOTX1 (*n* = 10 per group). **F** Insulin secretion in islets from T2D donors treated with KOTX1 as in (**E**) (*n* = 5 per group). **G** Immunofluorescence for Insulin, ALDH1A3, and PDX1 in *db/db* mice treated with KOTX1. Representative immunofluorescence images of *n* = 5 mice per group. Scale bars: 50 μM. All data are expressed as means ± SEM. Two-way ANOVA with multiple comparison test was used for statistical analysis for (**A**–**F**). Source data are provided as a Source Data file.

Data presented thus far show that diet-induced or genetic decreases of ALDH1A3 expression enhance β-cell function and maturity. We next tested whether acute ablation of ALDH1A3 activity through pharmacological inhibition of ALDH1A3 can produce similar effects. Whereas covalent, pan-ALDH inhibitors such as n,n,DEAB inhibit ALDH1A3 in vitro, there are no currently known ALDH1A3 selective inhibitors optimized to effectively inhibit ALDH1A3 in vivo. KOTX1 is a novel ALDH1A3 inhibitor that has been optimized for in vivo treatment^27^; KOTX1 is non-cytotoxic (Supplementary Fig. 3A) and reversible (Supplementary Fig. 3B), and it exhibits low nanomolar inhibition constants for ALDH1A3 (cellular IC_50_ 5.1 nM) (Fig. 3A, B and Supplementary Fig. 3C). Importantly, KOTX1 does not inhibit the closely related ALDH1A1 or ALDH2 isoforms (Supplementary Fig. 3D), which have been implicated in various metabolic and neurological pathways^28^. To examine the ability of KOTX1 to inhibit ALDH1A3 activity in vivo, we administered KOTX1 at 40 mg/kg/day to *db/db* animals by oral gavage for 1 week and isolated islets to measure ALDH1A3 activity using AldeRed^23^. Islets from vehicle-treated *db/db* mice showed elevated AldeRed activity (Supplementary Fig. 4A) that was completely abolished upon the addition of 10 uM KOTX1 to the AldeRed assay buffer (Supplementary Fig. 4B). In ex vivo culture, islets from KOTX1-treated *db/db* mice increased insulin secretion in response to glucose compared to islets from vehicle-treated controls (Supplementary Fig. 4C).

These data suggest that ALDH1A3 inhibition can have a disease-modifying effect in T2D models. To test this hypothesis, we compared the effect of KOTX1 on reversibility of β-cell dysfunction and dedifferentiation. We administered KOTX1 by oral gavage to 5-month-old diabetic *db/db* mice at 40 mg/kg/day for 4 weeks. We next performed IGTTs and found that KOTX1-treated *db/db* mice had improved glucose tolerance (Fig. 3C and Supplementary Fig. 4D). Importantly, plasma insulin levels increased in KOTX1- vs. vehicle-treated mice upon refeeding (Fig. 3D and Supplementary Fig. 4E). This is consistent with the results of the pair-feeding and genetic *β-Aldh1a3 KO_db/db* studies and indicates that KOTX1 improves β-cell function. We found no changes in body weight or food intake between KOTX1- and vehicle-treated *db/db* mice (Supplementary Fig. 4F, G). It is however possible that these effects can be partly accounted for by KOTX1 acting in other tissues. Thus, to analyze the direct effect of KOTX1 on β-cells, we examined islets from *db/db* mice or human diabetic donors. In both instances, treatment with KOTX1 resulted in ~50–150% increase of glucose-stimulated insulin secretion, respectively (Fig. 3E, F and Supplementary Fig. 4H). Immunohistochemistry further showed increased PDX1 and insulin (Fig. 3G), and both FACS and Immunohistochemistry analyses demonstrated decreased ALDH1A3 activity and expression, respectively (Fig. 3G and Supplementary Fig. 4I), supporting the notion that in vivo KOTX1 treatment restores β-cell function and maturity.

**Fig. 4:**
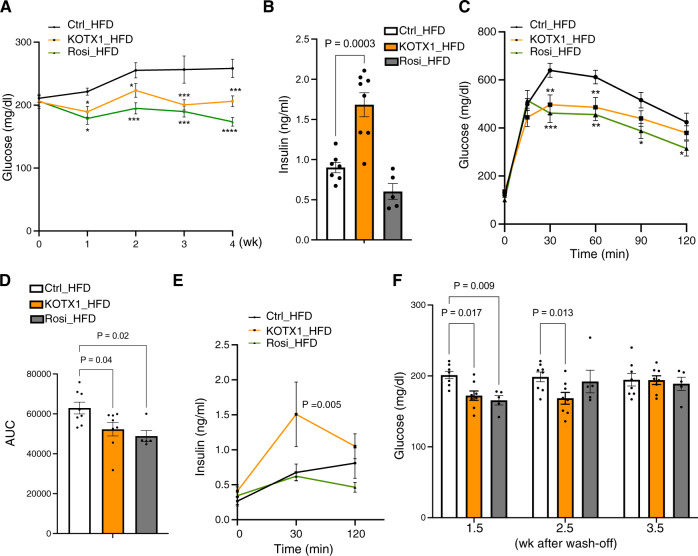
KOTX1 treatment in DIO model. **A** Non-fasting glucose in male DIO mice treated with KOTX1-, Rosi- or control for 4 weeks. Data are expressed as means ± SEM for *n* = 8, 8, or 5 for vehicle-control, KOTX1 or Rosi-HFD. Two-way ANOVA with multiple comparison test was used for statistical analysis. The *P* value was 0.036, 0.036, 0.0003, or 0.0008, for 1, 2, 3 or 4 weeks in comparison of KOTX1 with vehicle. In comparison of Rosi with vehicle, the p value was 0.015, 0.0007, 0.0002, or 0.00001, for 1, 2, 3 or 4 weeks of treatment. *P < 0.05, **P < 0.01, ***P < 0.001, ****P < 0.0001. **B** plasma insulin in male DIO mice treated with KOTX1-, Rosi- or control for 4 weeks. Data are expressed as means ± SEM for *n* = 7, 8, or 5 for vehicle-control, KOTX1 or Rosi-HFD. One-way ANOVA with multiple comparison test was used for statistical analysis. **C** IPGTT in male DIO mice treated with KOTX1-, Rosi- or control for 4 weeks. Data are expressed as means ± SEM for *n* = 8, 8, or 5 for vehicle-control, KOTX1 or Rosi-HFD. Two-way ANOVA with multiple comparison test was used for statistical analysis. The *P* value was 0.002 or 0.007 at 30 or 60 min in comparison of KOTX1 with vehicle. In comparison of Rosi with vehicle, the p value was 0.0007, 0.0032, 0.0169, or 0.0467 at 30, 60, 90 or 120 min. *P < 0.05, **P < 0.01, ***P < 0.001, ****P < 0.0001. **D** Quantification of areas under the curve for the IPGTT experiments in (**C**). Data are expressed as means ± SEM for *n* = 8, 8, or 5 for vehicle-control, KOTX1 or Rosi-HFD. One-way ANOVA with multiple comparison test was used for statistical analysis. **E** Plasma insulin during IPGTT. Data are expressed as means ± SEM (*n* = 7, 8, or 5 for vehicle-control, KOTX1 or Rosi-HFD). Two-way ANOVA with multiple comparison test was used for statistical analysis. **F** Non-fasting glucose in KOTX1-, Rosi- or vehicle-treated HFD fed during washout experiments after 4-week treatment. Data are expressed as means ± SEM for *n* = 8, 8, or 5 for vehicle-control, KOTX1 or Rosi-HFD. Two-way ANOVA with multiple comparison test was used for statistical analysis. Source data are provided as a Source Data file.

Next, we extended our studies to a model of diet-induced obesity (DIO) that more closely resembles clinically relevant T2D. To avoid the potential impact of daily gavaging in this diet-dependent model, we formulated KOTX1 into the high-fat diet (HFD) at a daily equivalent dose of 40 mg/kg (270 ppm). Pharmacokinetic analysis of serum from mice exposed to this diet for 5 days conducted at peak (nighttime) and trough consumption (afternoon) confirmed that this formulation resulted in sufficient exposure to KOTX1 (Supplementary Fig. 5A). Plasma KOTX1 levels were greater than 10-fold higher than the cellular IC_50_ even at trough conditions (Supplementary Fig. 5B), indicating that this dose effectively inhibited ALDH1A3 throughout the course of the experiment (Supplementary Fig. 5B). We fed 8-week-old mice HFD for 8 weeks, then randomized mice to either control diet, a diet containing rosiglitazone (positive control) or the diet with KOTX1 (KOTX1_HFD) for 4 weeks and monitored glucose levels throughout. Starting at week 1, we observed lower glucose in KOTX1- treated mice compared to the vehicle control (Fig. 4A). This effect was maintained throughout treatment, whereas vehicle-treated mice (Ctrl-HFD) showed gradually increasing glucose levels (Fig. 4A). We found no changes in weight or food intake in the KOTX1 diet compared to vehicle and Rosi diets (Supplementary Fig. 5B, C). While both KOTX1 and Rosi lowered glucose, only KOTX1 increased *ad lib* and refed insulin levels (Fig. 4B and Supplementary Fig. 5D). These data are consistent with the expectation that KOTX1 improves β-cell function as measured by insulin secretion, while rosiglitazone promotes insulin sensitivity. Furthermore, KOTX1 enhanced glucose tolerance as compared to Ctrl-HFD mice (Fig. 4C, 4D). Again, only in KOTX-HFD were insulin levels elevated during the IPGTT (Fig. 4E). Lastly, we investigated the persistence of the glucose-lowering effect of KOTX1. KOTX1–HFD mice maintained lower glucose levels for ~3 weeks following the withdrawal of KOTX1 (Fig. 4F), suggesting that there is a long-lasting recovery of β-cell function following ALDH1A3 inhibition. These findings are consistent with *β-Aldh1a3 KO_db/db* and pair-fed *db/db* studies (Figs. 1 and 2) and support the conclusion that inhibiting ALDH1A3 improves β-cell function.

### Cellular and molecular pathways of ALDH1A3 function in β-cells

**Fig. 5:**
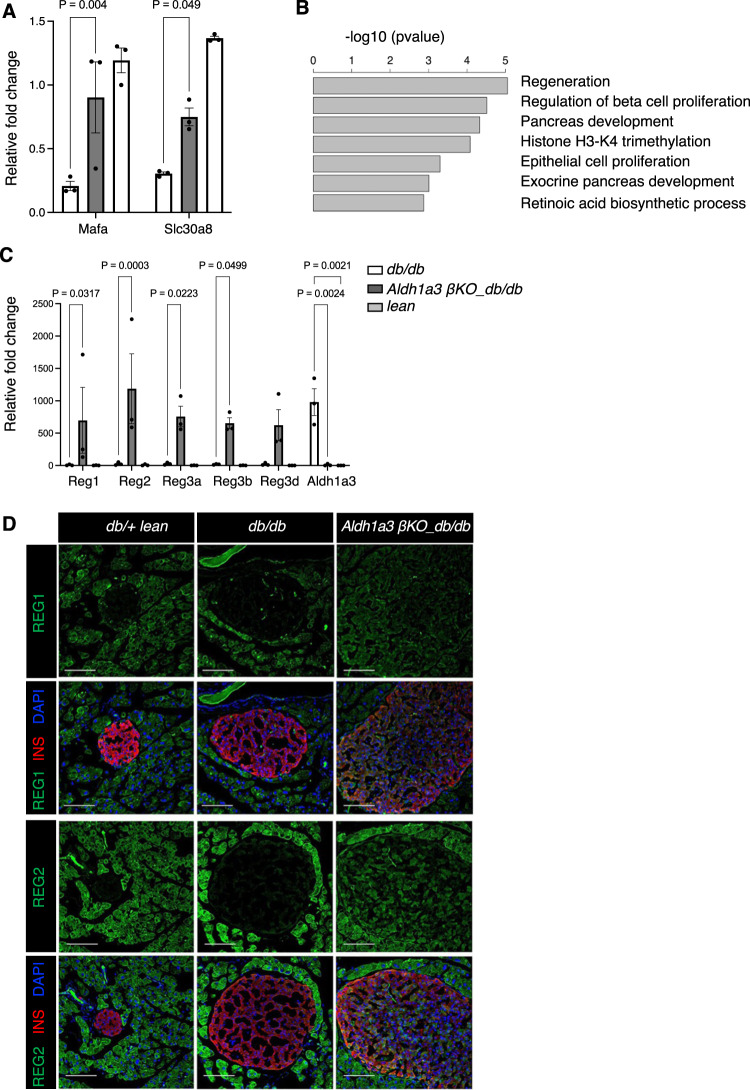
RNA expression pathways in Aldh1a3 knockout β-cells. **A** qPCR analyses of selected genes using sorted β-cells from *β-Aldh1a3 KO_db/db*, lean or *db/db* mice. Results were normalized by the expression of the 18S ribosomal subunit RNA and expressed as fold changes relative to expression levels in lean control. Data are expressed as means ± SEM for *n* = 3 biologically independent samples per group. Two-way ANOVA with multiple comparison test was used for statistical analysis. **B** GO terms of enriched genes in ALDH1A3 knockout β-cells. **C** qPCR analyses as in (**A**). Data are expressed as means ± SEM for *n* = 3 biologically independent samples per group. Two-way ANOVA with multiple comparison test was used for statistical analysis. **D** Co-immunostaining of REG1 or REG2 (green), insulin (Red) and DAPI (blue) in *db/+* (left), *db/db* (middle) or *β-Aldh1a3 KO_db/db* (right) pancreata. Representative immunofluorescence images of *n* = 4 mice per group. Scale bars: 100 μM. Source data are provided as a Source Data file.

To understand the mechanism by which ALDH1A3 affects β-cell function and maturity, we performed RNA-seq on sorted β-cells from *β-Aldh1a3 KO_db/db* and two control groups, *db/+* and *db/db* mice (Fig. 5A–C and Supplementary Fig. 6A). Hierarchical cluster analysis revealed that the gene profiles of *Aldh1a3* KO_β-cells are more similar to those of *db/+* lean mice than those of *db/db* (Supplementary Fig. 6B). This further supports our notion that inhibiting Aldh1a3 in *db/db* recovered the signature of β-cell identity. When we compared differentially expressed (DE) genes between *Aldh1a3* KO_*db/db* and *db/+* β-cells, and between *db/db* and *db/+* β-cells (Supplementary Data 1), we detected increases in gene expression involved in insulin secretion, glucose response, and cell surface receptor signaling, including MafA, Slc30a8, and Npr1^29,30^ commonly found in *Aldh1a3* KO and *db/+* β-cells compared to *db/db* (Supplementary Data 1). qPCR analyses further support a significant increase of MafA and Slc30a8 in *Aldh1a3* KO and *db/+* β-cells compared to *db/db* β-cells (Fig. 5A). Given that *β-Aldh1a3 KO_db/db* mice are still exposed to the insulin resistance imposed by the *Lepr* mutation, we were interested in understanding how *Aldh1a3* KO β-cells respond to sustained metabolic stress. To gain insight into this process, we performed Gene ontology (GO) analysis on DE genes in *Aldh1a3* KO β-cells compared to *db/+*, and in *db/db* β-cells compared to *db/+* controls (Supplementary Fig. 6A). This analysis showed significantly enriched GO terms for regeneration, pancreas development, and pancreatic β-cell proliferation (Fig. 5B and Supplementary Fig. 6C) in *Aldh1a3* KO β-cells. Notably, the Regenerating islet-derived (Reg) gene family (Reg1, Reg2, Reg3a, Reg3b, Reg3d, and Reg3g)^31,32^ showed higher expression in *Aldh1a3* KO β-cells (Fig.5C). Reg1 expression increases in replicating, regenerating, or hyperplastic islets but not in healthy or diabetic islets^33^, and has been proposed as a marker of replicating β-cells^34^. Administration of recombinant Reg1 or Reg3δ (INGAP) promotes islet proliferation and alleviates diabetes^35,36^.

**Fig. 6:**
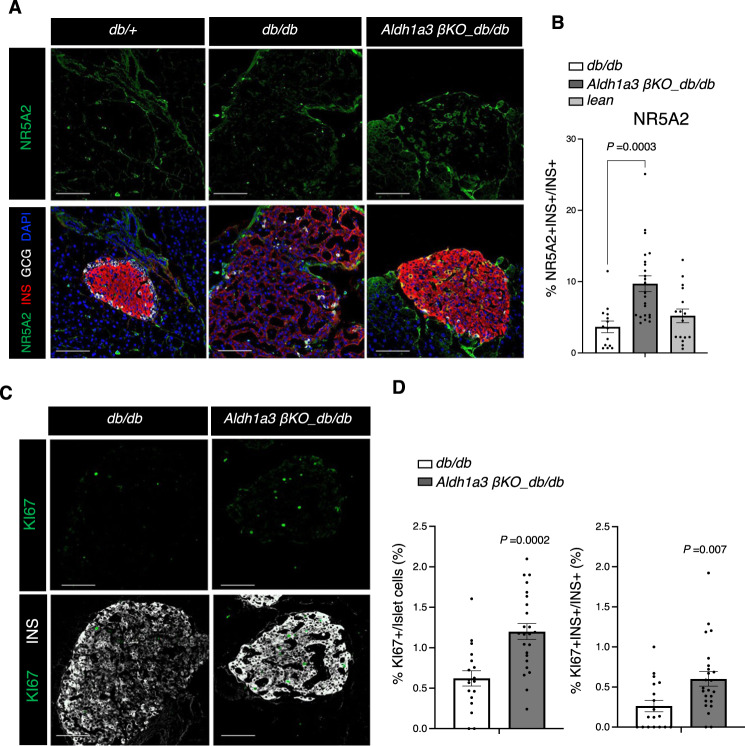
β-cell proliferation in *β-Aldh1a3 KO_db/db*. **A** Co-immunostaining of NR5A2 (green), insulin (Red), GCG (White) and DAPI (blue) in *db/+* (left), *db/db* (middle), or β-Aldh1a3 KO_*db/db* (right) pancreata. Scale bars: 100 μM. **B** Ratio of NR5A2-positive cells in INS+/GCG- β-cells. All data are expressed as means ± SEM. *N* = 14, 23 or 16 islets from 4 biologically independent samples per group. One-way ANOVA with multiple comparison test was used for statistical analysis. **C** Co-immunostaining of KI67 (green) and INS (white) in *db/db* or *β-Aldh1a3 KO*_*db/db* pancreata. Scale bars: 100 μM. **D** Ratio of KI67-positive cells to total islet cells (left) or INS+/GCG- cells (right). All data are expressed as means ± SEM. *N* = 18 or 24 islets from 4 biologically independent samples per group. Two-tailed paired *t*-test was used for statistical analysis. Source data are provided as a Source Data file.

We examined Reg expression by immunostaining. REG1 and REG2 were expressed exclusively in exocrine cells of lean or *db/db* controls, whereas in *β-Aldh1a3 KO_db/db* they were readily evident in islets (Fig. 5D). In contrast, REG3D was found in a subset of endocrine cells in control animals, but their number increased in *β-Aldh1a3 KO_db/db* pancreata (Supplementary Fig. 6D). In addition, RNA in situ hybridization showed increased *Reg1* expression in islets of *β-Aldh1a3 KO_db/db* but not *db/db* controls (Supplementary Fig. 7A). Spleen tissue in the same section served as a negative control (Supplementary Fig. 7B). Furthermore, we confirmed increased expression of NR5A2 (LRH-1) in sorted β-cells of *β-Aldh1a3 KO_db/db* mice (Supplementary Fig. 7C). NR5A2 is a regulator in early pancreas development^37^, gut cell proliferation and regeneration^38^, and stem cell pluripotency^39^. Immunohistochemistry further demonstrated increased numbers of NR5A2-expressing β-cells in *β-Aldh1a3 KO_db/db* compared to *db/db* controls (Fig. 6A, B).

Given the presumptive role of REG(s) and NR5A2 as well as the GO analyses suggestive of increased cell proliferation with enriched Ki67 gene (Supplementary Fig. 6C), we evaluated the cell proliferation marker, Ki67, by immunostaining. The number of Ki67-positive islet cells increased significantly in *β-Aldh1a3 KO_db/db* (Fig. 6C, D), including a twofold increase of Ki67+ β-cells. In contrast, we did not observe differences in apoptosis, as assessed by cleaved caspase-3 immunohistochemistry (Supplementary Fig. 7D). We further examined whether REG2- and Ki67-expressing cells increased in KOTX1-treated *db/db* mice. Consistent with studies in the conditional knockout model, KOTX1 increased REG2 and Ki67 expression (Supplementary Fig. 8A–C). In addition, we observed an increase of MafA, Slc30A8, and Gipr1 mRNA expression in KOTX1- *vs*. vehicle-treated *db/db* mice (Supplementary Fig. 8D). These findings suggest that genetic or pharmacologic ALDH1A3 inhibition also promotes β-cell proliferation, at least in the context of the *db/db* mutation.

### Retinoic acid signaling in β-cells

**Fig. 7:**
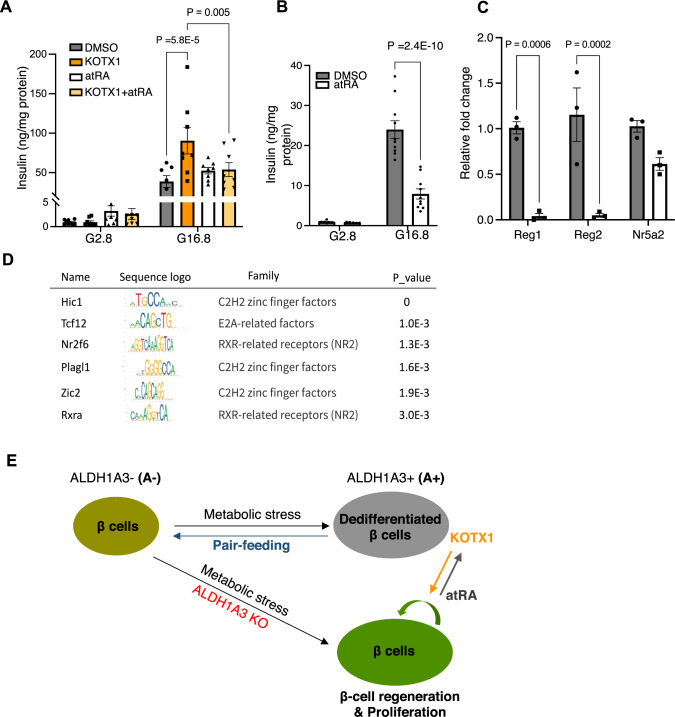
RA signaling and β-cell dysfunction. **A** Insulin secretion in islets from *db/db* mice following 3-day treatment with 10 μM KOTX1 or/and 50 nM atRA. Data are expressed as means ± SEM (*n* = 7 or 8 per condition). Two-way ANOVA with multiple comparison test was used for statistical analysis. **B** Insulin secretion assay as (**A**) using islets from *β-Aldh1a3 KO_db/db* mice. Data are expressed as means ± SEM (*n* = 10 per condition). Two-way ANOVA with multiple comparison test was used for statistical analysis. **C** qPCR analyses after atRA treatment of islets from *β-Aldh1a3 KO*_*db/db* mice. Results expressed as fold changes relative to expression levels in DMSO control. Data are expressed as means ± SEM for *n* = 3 biologically independent samples per group. Two-way ANOVA with multiple comparison test was used for statistical analysis. **D** Transcription factor motif analyses on DE gene promoters. **E** Schematic model of the effect of diet, genetic, or pharmacological ALDH1A3 inhibition in β-cells. Source data are provided as a Source Data file.

The canonical substrate of ALDH1A3 is retinaldehyde, which is converted to retinoic acid to activate nuclear retinoic acid (RA) signaling via binding the RAR receptors^40^. If the benefit of genetic or pharmacological Aldh1a3 inhibition were due to the suppression of RA signaling in diabetic β-cells, then treatment with all-trans RA (atRA) should circumvent the effects of KOTX1. To test this hypothesis, we treated islets from *db/db* mice with both atRA and KOTX1. When diabetic islets were treated with KOTX1, insulin expression increased, while co-treatment with atRA, abolished the KOTX1-mediated increase of insulin secretion (Fig. 7A). AtRA decreased insulin secretion (Fig. 7B), and dramatically reduced Reg expression in islets from *Aldh1a3* KO_*db/db* mice (Fig. 7C). These findings are consistent with the possibility that inhibition of RA signaling contributes to the mechanism of enhanced β-cell function in KOTX1-treated or ALDH1A3-deficient *db/db* mice. AtRA treatment had no effect in normal islets (Supplementary Fig. 8E), consistent with previous studies of ALDH1A3 overexpression in MIN6 cells^22^. These data show that RA treatment is sufficient to decrease insulin secretion in diabetic islets but not in normal islets, implying that additional factors contribute to diabetic phenotype.

To investigate potential drivers of the cellular regeneration pathway, we performed a motif analysis of transcription factors (TFs) that can bind to the promoters of differentially expressed genes, including Reg genes. We identified HIC1, TCF12, NR2F6 and RXRα as top regulators (Fig. 7D). Interestingly, expression of these TFs in diabetic islets is either controlled by RA signaling (HIC1)^41^, or dependent on interactions with RARs/RXRs to regulate target genes (NR2F6)^42,43^. For instance, HIC1 has been identified as an atRA-regulated gene in enterochromaffin cells^44^. Consistent with this, we found decreased HIC1 expression (Supplementary Fig. 8F) and increased proliferation in *Aldh1a3* KO β-cells (Fig. 6), suggesting HIC1 as a potential co-player of RA signaling in diabetic context.

## Discussion

In this study, we report that activation of ALDH1a3 expression in pancreatic β-cells is a reversible pathogenetic mechanism of β-cell dysfunction whose inhibition can be leveraged for treatment purposes. We have shown in previous work that this isoform of ALDH is normally not expressed in β-cells, and that its activation occurs in many mouse models of diabetes as well as in patients suffering from the disease^9,22^. We show that ALDH1A3 activation impairs insulin secretion and is associated with a loss of cellular differentiation features, and that these effects can be mimicked by the enzymatic product of ALDH1A3 activity, RA, presumably in an RAR/RXR-dependent fashion.

Approaches to treat β-cell failure can be subdivided into two categories: increasing cell number or promoting insulin secretion. The former has been pursued by transplantation of cadaver islets^45^ or stem cell-derived β-cells^46^ in patients requiring immune suppression for organ transplant. Stimulation of β-cell proliferation^47^ and inhibition of β-cell apoptosis have also been proposed^8,48^. Drugs that promote insulin secretion have been used for decades but are plagued by secondary failures^49^.

The discovery of dedifferentiation as a feature of β-cell failure raised the question of whether the process is reversible and, if so, whether it represents a druggable target^21^. Human studies in which low-calorie diets improve glucose homeostasis of diabetic patients corroborate the notion that the process is indeed reversible for a long time after disease onset^10–12,15^. However, the mechanism of this beneficial effect is unknown. Our findings demonstrate that dedifferentiated β-cells can revert to functional and mature β-cells, leading to improved insulin secretion and glucose control. This effect is mimicked by genetic and pharmacological ALDH1A3 ablation.

Unlike another target for reversal of β-cell failure, BACH2^21^, ALDH1A3 activation is an early event in the progression of β-cell failure^9,22,50^, and thus allows for earlier intervention to reverse disease progression. In addition, ALDH1A3 inhibition appears to activate a regeneration path, although whether this is limited to *db/db* mice or is a more general feature of this treatment remains to be determined. This is indicated not only by the reactivation of E-cadherin, whose expression correlates with β-cell proliferation and regeneration^51,52^, but also by changes in the expression of histone methyltransferases and demethylases, which are expected to raise H3K4me3 levels and result in an open chromatin structure, a distinctive feature of stem and progenitor cells. It is also consistent with recent studies indicating that activating β-cell proliferation by DYRK1A or/and TGFβ inhibitors increases β-cell differentiation markers, such as NKX6.1, GLP1R, and PCSK1^4,53^, with complementary epigenetic changes indicating that repressive (H3K27me3) and activating histone marks (H3K4me3) likely contribute to age-related control of β-cell proliferation^53–55^.

The KOTX1 IC_50_ is 10-fold lower in human A375 cells than in diabetic mouse islets, despite being similar in recombinant protein-based enzymatic assays in vitro. This may result from differences in cell number and mass between the two experiments affecting the amount of Aldefluor that can be oxidized and retained. In addition, KOTX1 is a substrate for some drug transporters. The Aldefluor assay buffer contains proprietary transporter inhibitors optimized for human cells that may facilitate KOTX1 efflux from mouse islets.

Our motif analysis and results with atRA indicate that ALDH1A3 acts at least partly through RA signaling. RA has distinct roles in pancreatic endocrine cell differentiation^56,57^ and the adult pancreas. The latter remains somewhat controversial, as transgenic mice with a dominant-negative RA receptor show an age-dependent decrease in insulin production^57^. However, these experiments were carried out in healthy β-cells that do not express ALDH1A3^22^, suggesting that the mutant RA receptor is acting through other nuclear receptors, for example, PPARs^58^. Two likely additional players in β-cell RA signaling identified in this paper and to be tested in future work are HIC1^41^ and NR2F6. In addition, we cannot rule out a role for non-canonical ALDH1A3 in β-cell function and maturation^59^, nor its function in glycolysis, which could lead to alterations in pyruvate supply to mitochondria.

In summary, the current work advances the field by showing the reversibility of β-cell dedifferentiation at the ALDH1A3 activation stage and providing proof-of-principle in preclinical models of its pharmacological inhibition as a treatment for diabetes.

## Methods

### Animals

All animal experiments were in accordance with NIH guidelines for Animal Care and Use, approved and overseen by Columbia University Institutional Animal Care and Use Committee (IACUC). Mice were housed under standard conditions on a 12-h light-dark cycle (lights on at 07:00 a.m.) and fed normal chow (PicoLab rodent diet 20, 5053; Purina Mills). For high-fat diet experiments, mice were fed a diet with 20% calories from carbohydrates, 20% from protein, and 60% from fat (D12492i; Research Diets). All experiments were performed in 16- to 20-week-old male and female mice, unless specified otherwise in the figure legend. Genotyping was as described^50^. To generate β-cell-specific *Aldh1a3* knockout mice, *Aldh1a3*^*Tm1a*^ mice (Mouse Biology Program at UC Davis) were crossed with FLP0 mice (Jackson Laboratories) to remove the FRT-flanked selection cassette, then bred with *RIP-Cre*^*herr*^:*Rosa26-lox-STOP-lox-tdTomato (R26R-tdT)* mice to obtain *RIP-Cre*^*herr*^*:Aldh1a3*^*fl/fl*^*: tdT*^*fl/+*^ mice. This line was backcrossed onto *db/db* (B6.BKS(D)-Lepr^db^/J, Jackson Laboratories #000697) to generate *β-Aldh1a3 KO_db/db* mice *(RIP-Cre*^*Herr*^*:Aldh1a3* ^*fl/fl*^*:tdT*^*fl/+*^*:Lepr*^*db/db*^). To generate *Aldh1a3-Cre*^*ert*^ mice for lineage-tracing experiments, tamoxifen-activable Cre (Cre^ert^) was knocked into the endogenous *Aldh1A3* locus, then the mutant allele was introduced into *Rosa26-lox-STOP-lox-YFP (R26R-YFP)* mice. Human endpoints for immediate euthanasia included lethargy with ruffled fur, hunched posture, reduced motility (inability to reach food and water), dehydration, abnormal respiration, significant weight loss (>20% of their body weight), or blood sugar >400 mg/dL for more than 2 weeks. CO_2_ euthanasia was performed in accordance with the AVMA Guidelines for Animal Euthanasia (2020 Edition).

### Metabolic analyses

We performed intraperitoneal glucose tolerance tests (IPGTT) (1 g/kg) after an overnight fast^60^. We measured serum insulin by ELISA (Mercodia #10-1247-01). KOTX1 was administered orally (40 mg/kg) to male *db/db* mice daily for 4 weeks. For HFD experiments, KOTX1 was added to the diet. Non-fasting glucose and insulin were assessed throughout the study. For fasting and refeeding experiments, animals were fasted for 16 h before measurement of glucose and insulin unless otherwise indicated.

### Immunohistochemistry

We performed immunohistochemistry as described^6^. We applied heart-perfused fixation with 4% paraformaldehyde (PFA). Pancreata were incubated in 4% paraformaldehyde (PFA) at 4 °C overnight, washed with ice-cold PBS three times, and placed in 30% sucrose overnight^16^. Tissue was embedded in Tissue-Tek optimal cutting compound (Sakura Finetek), frozen on dry ice, and cut into frozen 5-μm sections. We applied antigen retrieval to detect transcription factors (Nacalai USA Inc.). We used primary antibodies to INSULIN (A056401-2; Dako; 1:1000), GLUCAGON (G2654; Sigma-Aldrich; 1:1000), PDX1 (ab47308; Abcam; 1:100), E-cadherin (61018; BD Biosciences; 1:100), REG1 (AF1657; R&D systems; 1:100), REG2 (AF2035; R&D systems; 1:100), REG3d (MAB5678; R&D systems; 1:100), NR5a2 (PPH2325; R&D systems; 1:100), KI67 (GTX16667; Genetex; 1:100), MAFA (IHC-00352; Bethyl Laboratories; 1:100), Cleaved Caspase-3 (9661; Cell Signaling; 1:100), and ALDH1A3 (NBP2-15339; Novus Biologicals; 1:100) and Alexa Fluor–conjugated goat serum as a secondary antibody (Jackson ImmunoResearch Laboratories and Molecular Probes). The images were captured using a Zeiss LSM 710 confocal microscope using a 20× objective and analyzed using ZEN.

### RNAScope mRNA in situ hybridization assay

RNAscope was performed using the RNAscope 2.5 HD Detection Reagent RNAscope 2.0 High-Definition kit (Advanced Cell Diagnostics; 322350) combined with immunofluorescence according to the manufacturer’s instructions. A mouse Reg1 probe (511571; ACD) was used to detect Reg1 mRNA. Briefly, tissue sections were baked for 1 h at 60 °C, and treated with Pretreat 1 for 10 min at room temperature (RT). Target retrieval was performed for 15 min at 100–104 °C, followed by protease treatment for 15 min at 40 °C. Probes were then hybridized for 2 h at 40 °C followed by RNAscope amplification followed by red chromogenic detection.

### Human islet procurement

Human T2D islets were from the National Institutes of Health’s Integrated Islet Distribution Program (IIDP). Upon arrival, islets were plated at a density of 10,000 IEQ per 10-cm non-treated tissue culture dish (Corning, Corning, NY; cat. no. 430591) into 10 mL of islet culture medium (Prodo Labs, PIM(S), cat#PIM-CS001GMP), supplemented with 5 mL PIM(G) Glutamine/Glutathione (Prodo Labs, cat#PIM-G001GMP), and 5% PIM(ABS) Human AB Serum (Prodo Labs, cat#PIM-ABS001GMP), along with triple antibiotics, PIM(3X), which includes Ciprofloxacin (Ref 61–277-RF, 10 mg/1000 mL), Gentamycin (Sigma, G1272, 10 mg/1000 mL), and Amphotericin B (Omega, FG-70, 2500 mcgm/1000 mL). Islets were cultured for no longer than 1 week after arrival and the medium was replaced every 2 days. All islets from the IIDP are obtained from cadaver donors. Before providing the de-identified islets to established laboratories involved in islet research and registered with IIDP, the IIDP obtains informed consent for research purposes from donor relatives. Human subjects’ research at the NIH does not include research on de-identified specimens from deceased donors (NHSR). Therefore, no special permissions are required.

### Islet isolation

We isolated mouse islets by collagenase digestion^58^. Mice were fasted for 16 h before islets isolation which was used for FACS and RNA-Seq analyses. Briefly, the animal was euthanized in a CO_2_ chamber followed by cervical dislocation. The common bile duct was clamped with a hemostat near the liver, and 3 mL of 1 mg/mL cold Collagenase P (Sigma 11249002001) solution was injected into the hepatopancreatic ampulla to inflate the pancreas^50^. The excised pancreas was incubated at 37 °C with shaking for 16 min. Medium 199 was added to a final volume of 50 mL, and the mixture was centrifuged at 200 × g for 2 min at 4 °C. The pellet was resuspended with 10 mL of histopaque, and 10 mL of Medium 199 was layered on top of the histopaque followed by centrifugation at 1200 × g for 20 min at 4 °C. Islets at the interface of the histopaque and Medium 199 were collected and washed twice with Medium 199 containing 10% FBS (Sigma-Aldrich F2442). Islets were handpicked into RPMI 1640 medium (ThermoFisher 11150-067) containing 15% FBS (Sigma-Aldrich F2442) for further analysis.

### Glucose-stimulated insulin secretion

Islets were preincubated in KRBH buffer (10 mM HEPES pH 7.4, 140 mM NaCl, 1.5 mM CaCl2, 3.6 mM KCl, 0.5 mM NaH2PO4, 0.5 mM MgSO4, 2 mM NaHCO3, and 0.1% BSA) for 1 h at 2.8 mM glucose, followed by incubation in KRBH at 2.8, and 16.7 mM glucose for 1 h at 37 °C. At the end, we collected islets by centrifugation and assayed the supernatant for insulin secretion or lysates for insulin content by enzyme-linked immunosorbent assay (ELISA; Mercodia 10-1247-01). Insulin levels were normalized by protein concentration.

### Fluorescence-activated β-cell sorting

We sorted Aldh+ β cells as described^22^. Briefly, isolated islets were trypsinized to single cells and then incubated with the fluorescent ALDH substrate, AldeRed (Sigma, Cat. SCR150) for 1 h prior to flow cytometry. Thereafter, cells were loaded into a BD Influx sorter. We gated cells for YFP (green) and aldeRed (Red) fluorescence.

### Enzyme activity assays

KOTX1 was synthesized by WuXi apptec Co. with 98.2% purity as determined by LC-MS/^1^H NMR. For recombinant protein selectivity analysis of KOTX1, human ALDH1a1, ALDH1a3, ALDH2 were cloned from human reference DNA and mouse ALDH1a3 was cloned from mouse reference DNA into the pET bacterial expression system. Crude recombinant protein lysates were extracted from IPTG-induced bacterial culture. Recombinant protein activity assays were conducted as described^61^ except resazurin was added to 10 µM and diaphorase (Thermo fisher) to 200 U/mL. Reaction kinetics were monitored by detecting the production of resorufin by fluorescence at 590 nm. Reaction velocity was calculated ΔFl/Δt and normalizing rates to a DMSO control. For cell-based potency analysis, the Aldefluor assay was conducted according to the manufacturer’s suggestions with A375 cells (ATCC, cat. CRL-1619) suspended at 1 million cells/mL or diabetic mouse islet cells. KOTX1 was serially diluted into DMSO and then equivalent volumes were added to Aldefluor buffer to avoid DMSO effects on cell membrane permeability. The reversibility of KOTX1 is assessed using the Aldefluor assay in diabetic mouse islets with KOTX1 incubation for 1 h followed by wash-off KOTX1 for twice with the assay buffer.

### Cell cytotoxicity assays

We used the CellTiter-Glo 2.0 Cell Viability Assay (Promega) to examine the cytotoxicity of KOTX1 treatment at different concentrations (10 nM–10 uM) in pancreatic islet cells (50,000 or 100,000 cells) according to the manufacturer’s instructions.

### RNA measurements

We isolated RNA with the RNeasy Micro-kit (QIAGEN) and reverse-transcribed RNA using qScript cDNA SuperMix (Quanta). Quantitative polymerase chain reaction (qPCR) was performed using GoTaq qPCR Master Mix (Promega). The following are the PCR primer sequences: mReg1 (F: 5′-AACTTTGTGGCCTCTCTGATTA-3′, R: 5′-CACAGTTGTCATCCTTCCATTTC 3′), mReg2 (F: 5′-GTGGTACTACAGCTTCCAATGT-3′, R: 5′-GGCCCATGACTTGAAGAGAAA-3′), mReg3a (F: 5′-CAGAGTGGACAACTACCAAGAC-3′, R: 5′-AGTTACTCCACTCCCATCCA-3′), mReg3b (F: 5′-ACAGGAAACAGCTACCAATACA −3′, R: 5′-GGGTTCCTCTCCCAGTTAAAG-3′), mReg3d (F: 5′-AGTTTGACCACATTCCCATACA −3′, R: 5′-ATAGAAGGTCAGAGGGTCAGAG-3′), mNr5a2 (F: 5′-TTGAGTGGGCCAGGAGTAGT-3′, R: 5′-ACGCGACTTCTGTGTGTGAG-3′), mSlc30a8 (F: 5’- CCAGCACCGTCATGATCTTA-3’, R: 5’- ACTGCGAGGATGATCTCTTTC-3’), mGipr1 (F: 5’- GACGGAGGAACAGGTTGAAG-3’, R: 5’- GGAAACCCTGGAAGGAACTTAG-3’) and mHic1 (F: 5′-CTTCTAACTCGGCTCCTTGTC-3′, R: 5′-CAGCACACTCTCCCGATTT-3′). All other PCR primer sequences have been published^22^. Gene expression levels were normalized to 18s rRNA or β-actin using the ΔΔCT method and are presented as relative fold-change to control.

### RNA-sequencing

Poly-A RNA-Seq libraries were prepared using Illumina TruSeq chemistry and sequenced as PE100 (100 bp paired-end reads) on Illumina NovaSeq 6000. Gene expression quantification was performed using kallisto^62^ based on the GRCm38 mouse reference genome. Differentially gene expression analysis was performed using DESeq2^63^. For heatmap visualization, we prioritized the top 50 most upregulated genes in Aldh1a3 KO compared to db/db or upregulated genes in db/db compared to lean control, and visualized them in Supplementary Fig. 6B. Principal component analysis (PCA) was performed on kallisto normalized expression matrix using function prcomp in R package stats (version 3.5.0). Hierarchical clustering analysis was performed on kallisto normalized expression matrix using function hclust in R package stats (version 3.5.0).

### Motif analysis

The motif analysis searches for transcription factors (TFs) that potentially control the differentially expressed genes identified in Fig. 5A. We followed the workflow as previously described in ref. ^64^. Specifically, we (1) retrieved the genomic information of the differentially expressed genes from the Bioconductor R TxDb.Mmusculus.UCSC.mm10.knownGene annotation using the function genes in the Bioconductor R package GenomicFeatures; (2) identified the promoter regions of the differentially expressed genes using the function promoters in the Bioconductor R package GenomicRanges; (3) retrieved TF position frequency matrices (PFMs) that are documented in the Bioconductor R JASPAR2020 database using the function getMatrixSet in the Bioconductor R package TFBSTools; (4) retrieved the promoter sequences of the differentially expressed genes from the Bioconductor R BSgenome.Mmusculus.UCSC.mm10 database, and match such sequences with the TF PFMs using functions matchMotifs and motifMatches in the Bioconductor R package motifmatchr. By this means, we quantified the binding score between the differentially expressed genes and all the TFs documented in the JASPAR2020 database. To further quantify the confidence of such binding scores, we created TF-specific null models with 1000 independently generated random gene sets. Such gene sets contain the same number of genes as in Supplementary Fig. 6B (top panel). We prioritized TFs based on the *P* values calculated from such null models.

### Gene ontology analysis

The DE genes (log2 > 1.5, *P* < 0.05) between Aldh1a3 KO and lean control β-cells or between db/db and lean control β-cells were used for The gene ontology (GO) analysis using an online tool, PANTHER 17 (http://geneontology.org/). Specifically, we performed the analysis using mouse biological processes-related GO terms. All parameters were set to default when performing the online GO analysis.

### Statistics

The statistical analyses were performed using Prism 8.0 software (Graph Pad). ANOVA with Dunnett’s method was performed between the two groups. All results are presented as means ± standard error of the mean (SEM).

### Reporting summary

Further information on research design is available in the Nature Portfolio Reporting Summary linked to this article.

## Supplementary information

Supplementary Information

Description of Additional Supplementary Files

Supplementary Data 1

Reporting Summary

## Data Availability

RNA-Seq data have been deposited at the Gene Expression Omnibus (GEO) under accession number GSE218047. All other data that support the findings of this study are provided in the article or supplementary data. Source Data are provided with this paper.

## Source data

Source Data
