# Supplementary material for: Genetic and pharmacologic inhibition of ALDH1A3 as a treatment of β-cell failure
Source: Nat Commun. 2023 Feb 2;14:558. doi: 10.1038/s41467-023-36315-4 (PMC9895451; doi:10.1038/s41467-023-36315-4)
Supplement: Supplementary file 1 — Supplementary Information [file 41467_2023_36315_MOESM1_ESM.pdf]

Supplementary Fig. 1

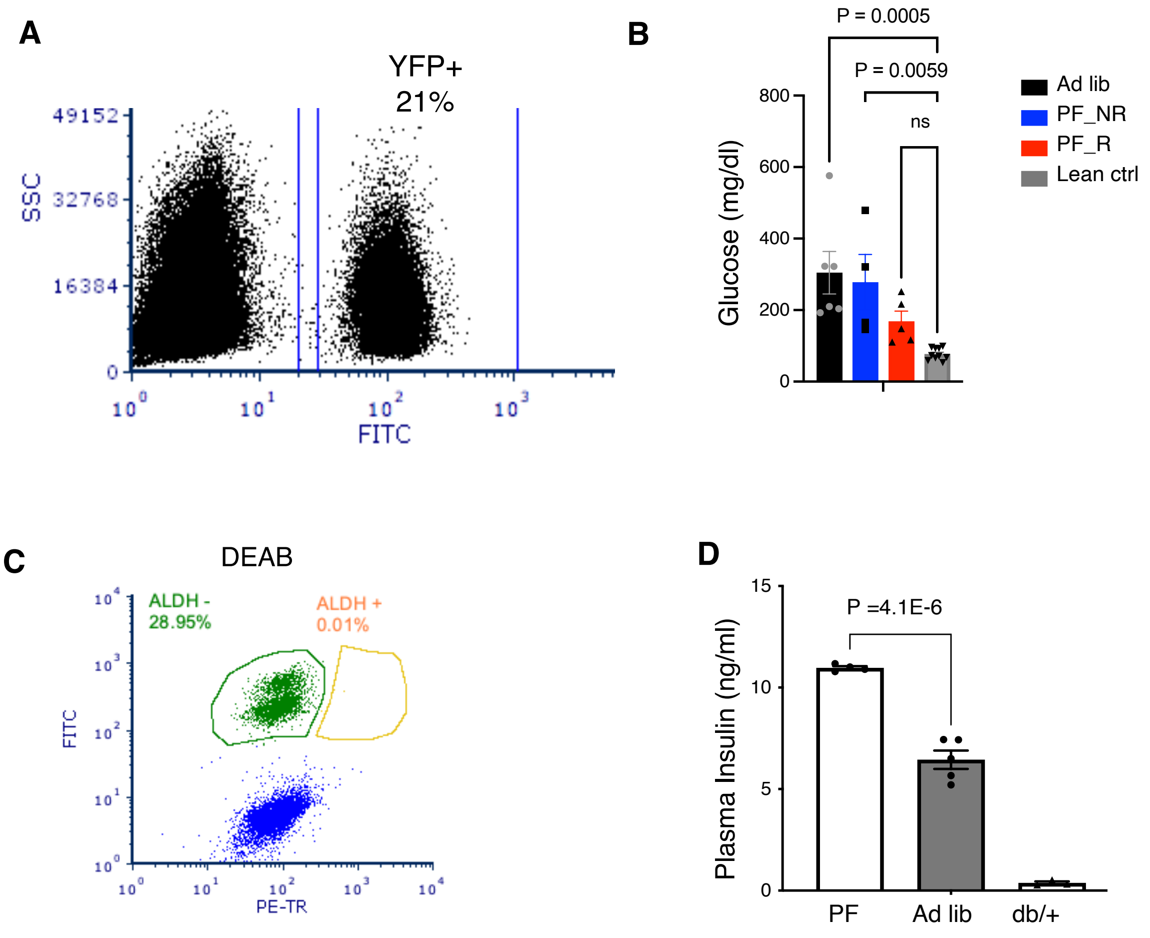

Supplementary Fig. 1 Pair-feeding in Aldh1a3-Cre^{ert}:YFP$^{fl/+}$ *db/db* mice

(A) FACS analyses of YFP+ cells in Aldh1a3-Cre^{ert}:YFP$^{fl/+}$ *db/db* mice after 5-day tamoxifen treatment. (B) Glucose levels after 16-hr fasting in pair-fed or *ad lib* Aldh1a3-Cre^{ert}:YFP$^{fl/+}$ *db/db* mice. Aldh1a3-Cre^{ert}:YFP$^{fl/+}$ db/+ mice were used as controls. (n = 6, 5, 4 or 10 for ad lib, PF_R (Pair-fed responder), PF_NR (Pair-fed non-responder or lean mice). Data are expressed as means ± SEM. One-way ANOVA with multiple comparison test was used for statistical analysis. (C) FACS analyses of DEAB-treated cells in AldeRed assays. (D) Fasting insulin measured by ELISA after two weeks of pair-feeding. (n = 5, 4, or 3 for ad lib, PF_R or lean mice). Data are expressed as means ± SEM. One-way ANOVA with multiple comparison test was used for statistical analysis.

Supplementary Fig. 2

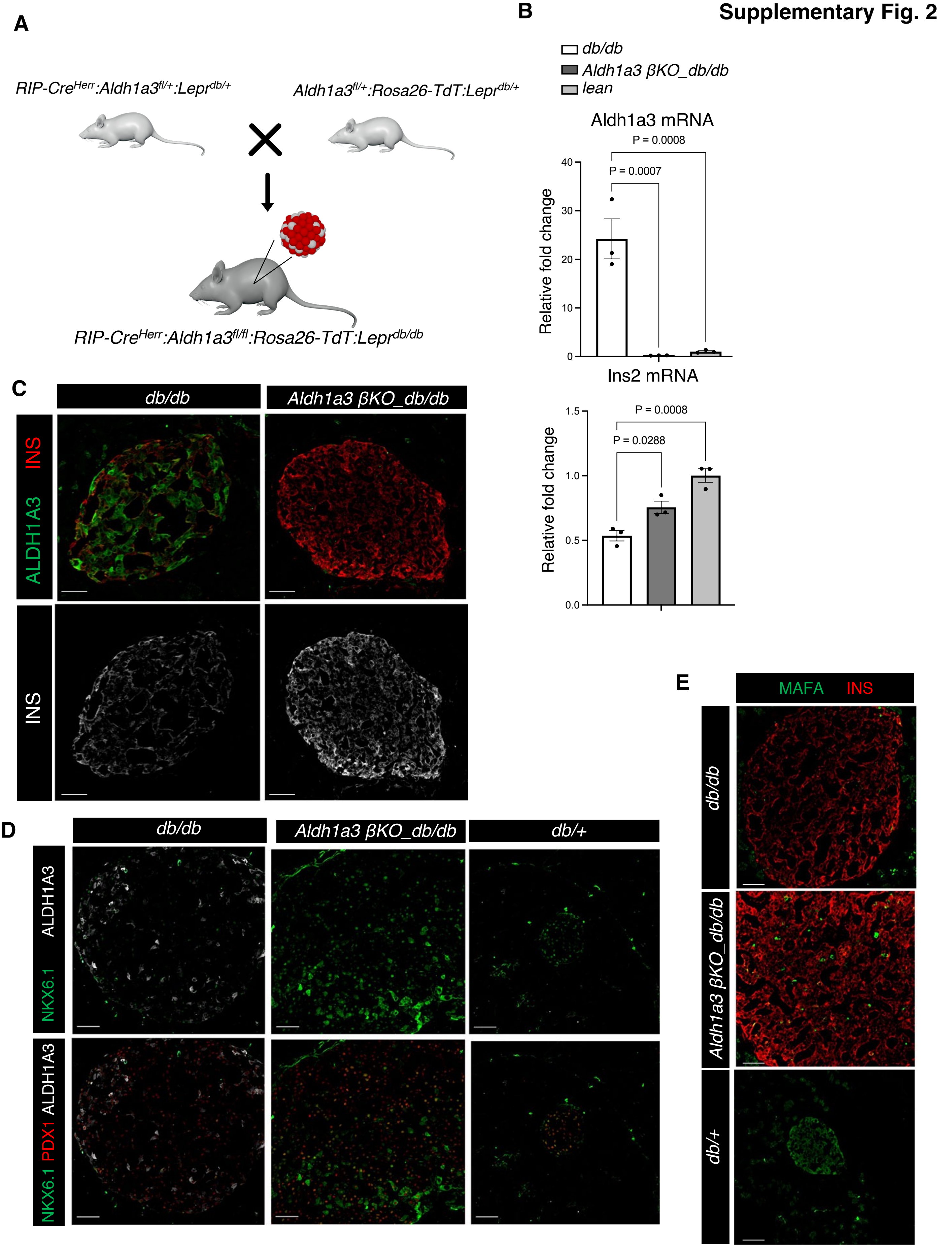

Supplementary Fig. 2 Generation of *β-Aldh1a3 KO_db/db* mice

(A) Breeding strategy to generate β-cell-specific Aldh1a3 knockout mice by crossing Aldh1a3^{Tm1a} with FLP0 mice to remove the FRT-flanked selection cassette followed by cross with *RIP-Cre^{herr}:Rosa26-lox-STOP-lox-tdTomato* (R26R-tdT) to obtain *RIP-Cre^{herr}:Aldh1a3$^{fl/fl}$:tdT^{fl}/+*. This line was backcrossed onto a *db/db* background to generate *β-Aldh1a3 KO_db/db* mice (RIP-Cre^{herr}:Aldh1a3$^{fl/fl}$:tdT^{fl}/$^{+}$:Lepr$^{db/db}$). (B) Aldh1a3 and insulin qPCR in sorted β-cells from mice of the indicated genotypes. Data are expressed as means ± SEM for n = 3 biologically independent samples. One-way ANOVA with multiple comparison test was used for statistical analysis. (C) Immunofluorescent staining of ALDH1A3 and INS in β-Aldh1a3 KO_db/db or db/db mice. Representative immunofluorescence images of n=3 mice per group. Scale bars: 50 μM. (D),(E) Immunofluorescent staining of β-cell markers, NKX6.1 (D) or MAFA (E). Representative immunofluorescence images of n=3 mice per group. Scale bars: 50 μM.

Supplementary Fig. 3

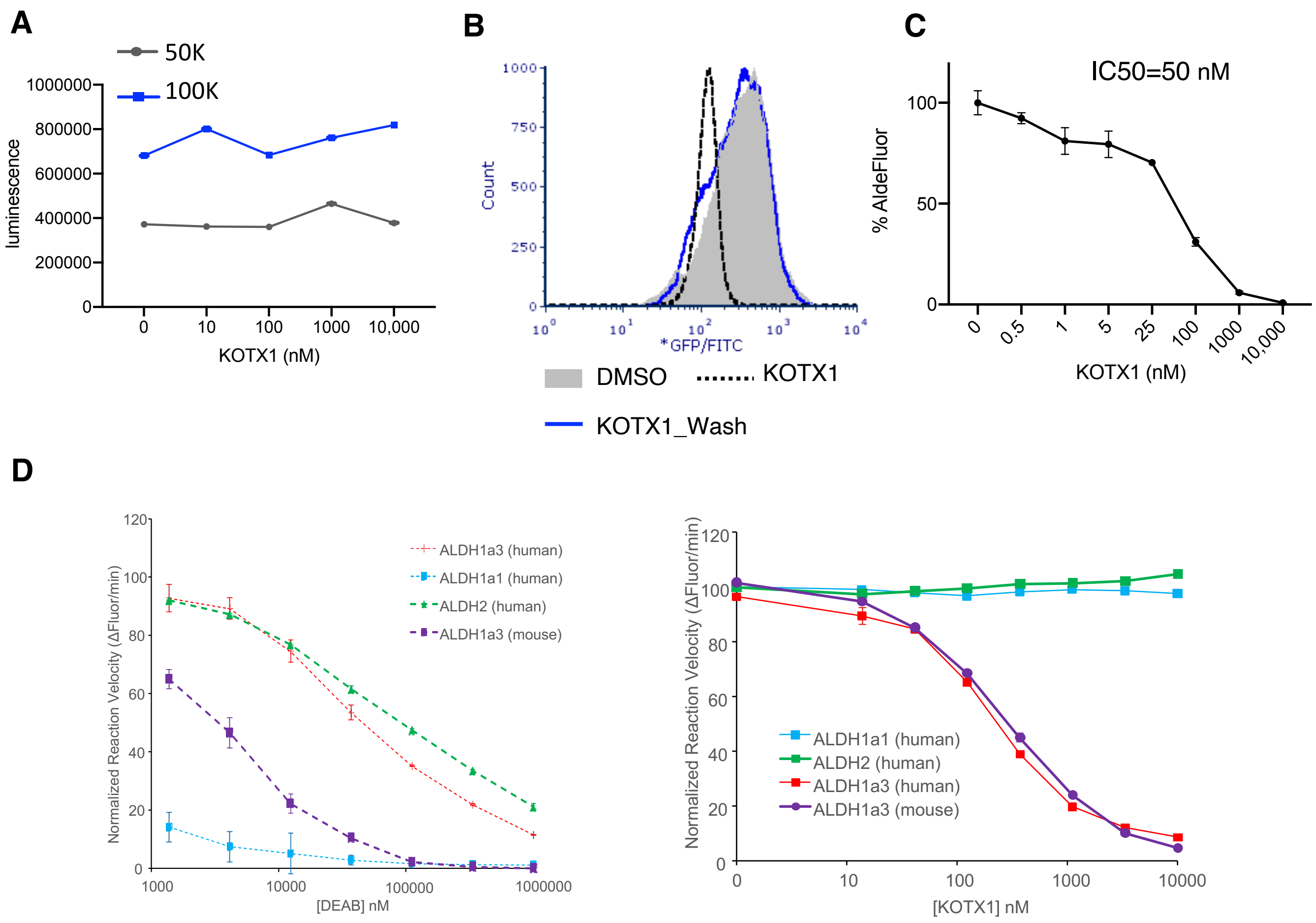

Supplementary Fig. 3 (A) Cell Titer-Glo Cell Viability Assay performed with different concentrations of KOTX1 using 50,000 or 100, 000 pancreatic islet cells. Data are expressed as means ± SEM for n = 2 biologically independent samples. (B) AldeFluor activity of *db/db* islets treated with DMSO or KOTX1, as well as KOTX1 washout. (C) Dose titration of KOTX1 using diabetic islets to assess AldeFlour activity. Data are expressed as means ± SEM for n = 2 biologically independent samples. (D) Dose titration enzymatic assays testing the inhibition of recombinant ALDH1a3 (mouse/human), ALDH1a1 (human) and ALDH2 (human) by either N,N Diethylaminobenzaldehyde (Left) or KOTX1 (Right). Data are expressed as means ± SEM for n = 3 biologically independent samples.

Supplementary Fig. 4

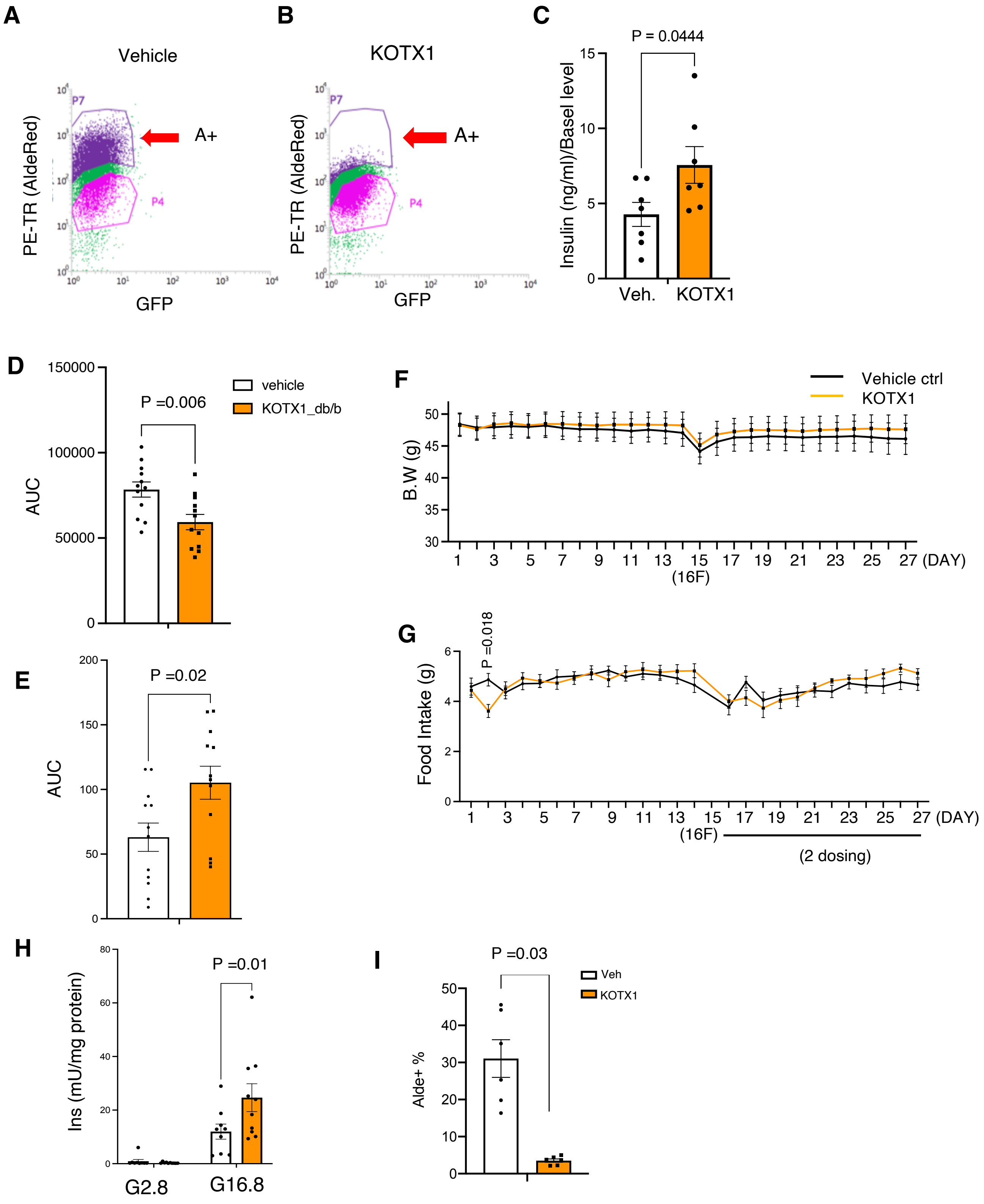

Supplementary Fig. 4 (A-B) 1-week treatment with ALDH1A3 inhibitor, KOTX1 effectively inhibits ALDH1A3 activity in islets of mice. (C) Insulin secretion in islets from KOTX1-treated *db/db* mice. Data are expressed as means ± SEM from 7 biologically independent samples per group. Two-tailed paired t-test was used for statistical analysis. (D) Quantification of areas under the curve for the IPGTT in KOTX1- or vehicle-treated *db/db* mice. Data are expressed as means ± SEM from 12 biologically independent samples per group. Two-tailed unpaired t-test was used for statistical analysis. (E) Quantification of areas under the curve for plasma insulin after refeeding as in (D). Data are expressed as means ± SEM from 12 biologically independent samples per group. Two-tailed unpaired t-test was used for statistical analysis. (F-G) Body weight (F) and daily food intake (G) during KOTX1 treatment. All data are expressed as means ± SEM for 12 biologically independent samples per group. Two-way ANOVA with multiple comparison test was used for statistical analysis. (H) Glucose-induced insulin secretion in islets from T2D donor #2. Data are expressed as means ± SEM for 9 or 10 experimental samples per group. Two-way ANOVA with multiple comparison test was used for statistical analysis. (I) The percentage of Alde+ cells in islets from db/db mice treated with vehicle or KOXT1 for four weeks. Data are expressed as means ± SEM from 6 biologically independent samples per group. Two-tailed paired t-test was used for statistical analysis. Source data are provided as a Source Data file.

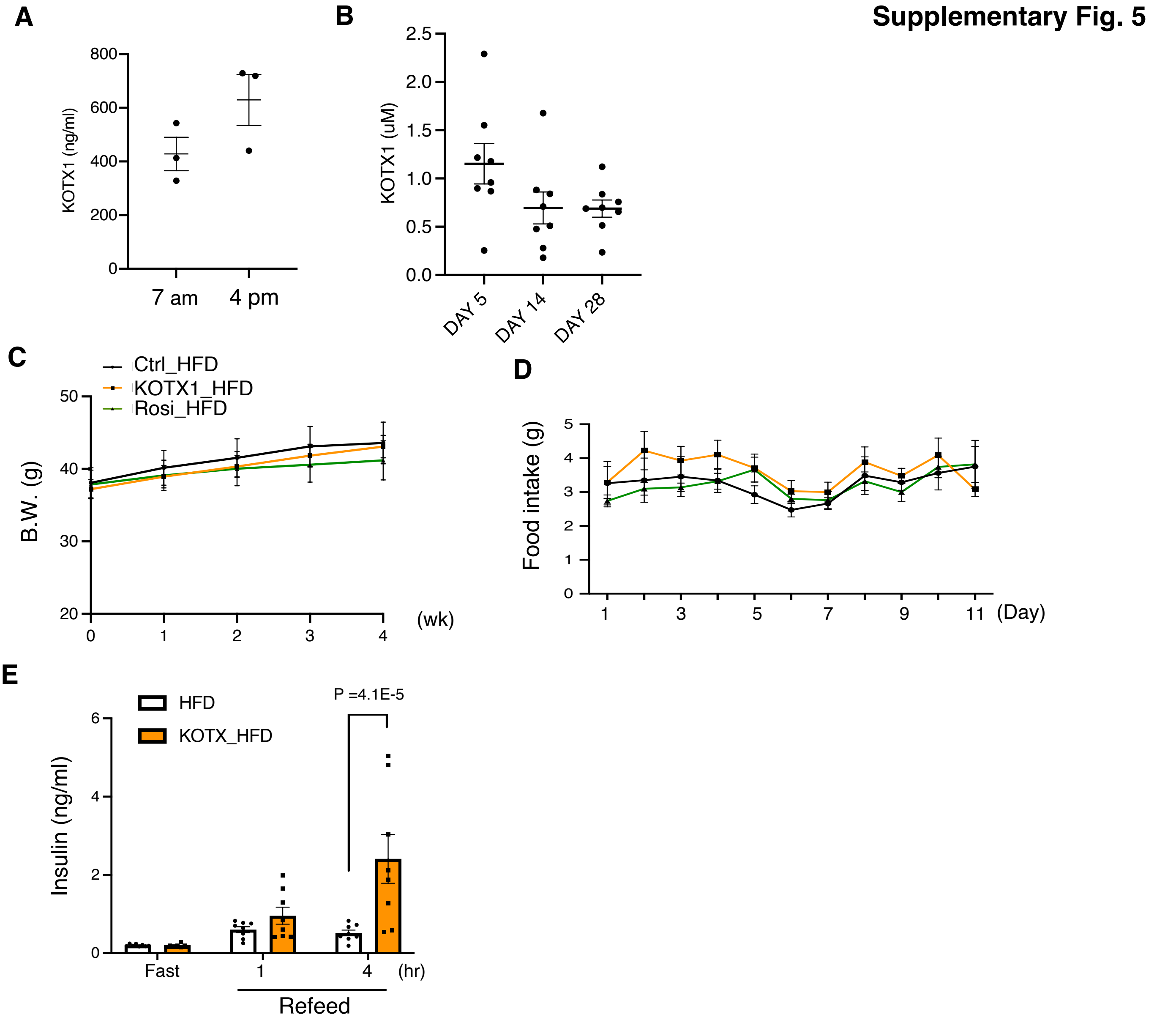

Supplementary Fig. 5 (A) Pharmacokinetics of HFD_KOTX1 in Male CD-1 mice on day 3 of treatment. Data are expressed as means ± SEM from 3 biologically independent samples per condition. (B) Pharmacokinetics of HFD_KOTX1 in DIO mice on day 5, day 14 or day 28. Data are expressed as means ± SEM from 8 biologically independent samples per condition. (C-D) Body weight (C) and daily food intake (C) in KOTX1_HFD-fed mice. Data are expressed as means ± SEM for n = 8, 8, or 5 for vehicle-control, KOTX1 or Rosi-HFD. Two-way ANOVA with multiple comparison test was used for statistical analysis. (E) Insulin levels in fasted and refed mice 2 weeks after treatment. Data are expressed as means ± SEM for n = 8, or 8 for vehicle-control or KOTX1-HFD. Two-way ANOVA with multiple comparison test was used for statistical analysis. Source data are provided as a Source Data file.

Supplementary Fig. 6

A

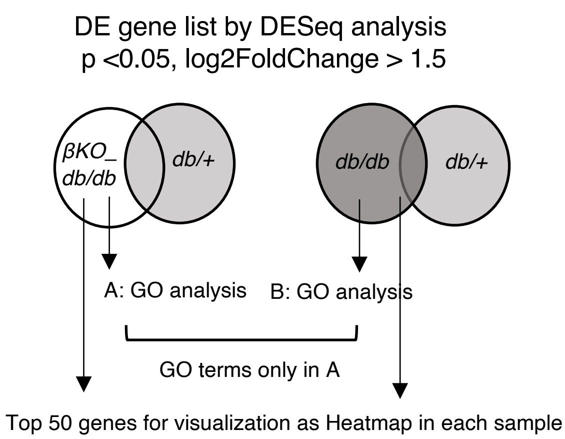

B

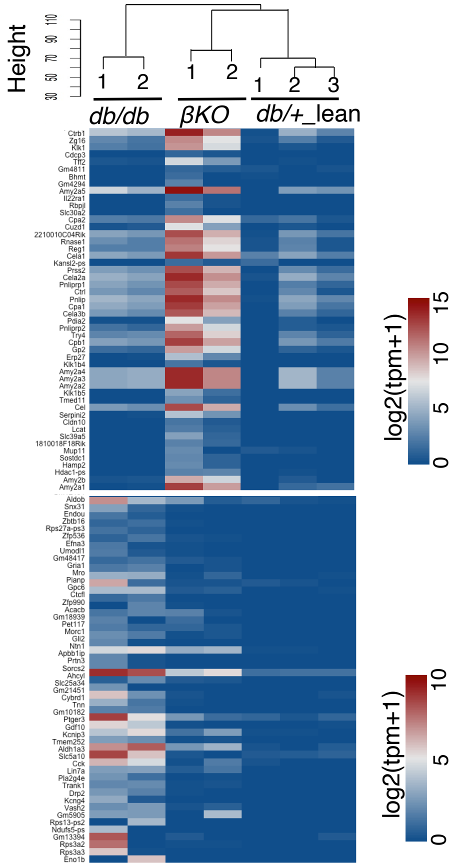

C

GO Term	FDR	Genes
Regeneration	8.61E-04	Apoa1, Cd9, Reg1, Tnr, Nefh, Gata4, Cdkn1a, Igf1, Lamb2, Sulf2
		Cebpb, Plg, Large1, Mapk8ip3, Jun, Igsf10
Regulation of beta cell proliferation	2.28E-03	Nupr1, Reg1, Irs2, Errfi1, Igf1, Reg2, Nr1d1
Pancreas development	3.26E-03	Igf1r, Cela1, Reg1, Ptf1a, Cdk6, Igf1, Sox4, Hnf1a, Nr5a2, Aldh1a7, Neurog3, Ildr2, Ccdc40
Histone H3-K4 trimethylation	5.22E-03	Kmt2d, Zfp335, Kmt2a, Tet3, Tet2, Ncoa6, Setd1a
Epithelial cell proliferation	2.11E-02	Apoa1, Kdr, Cav2, Igfbp3, Rps6ka1, Irs2, Notch2, Col18a1, Serpinb1a, Fermt1
		Igf1, Epha2, Mki67, Cebpb, Ovol1, Tbx2, Col8a2, Esr1, Apc, Kit
Exocrine pancreas development	3.63E-02	Igf1r, Cela1, Ptf1a, Igf1
Retinoic acid biosynthetic process	4.72E-02	Rdh9, Aldh1a3, Aldh1a7, Aldh1a1

D

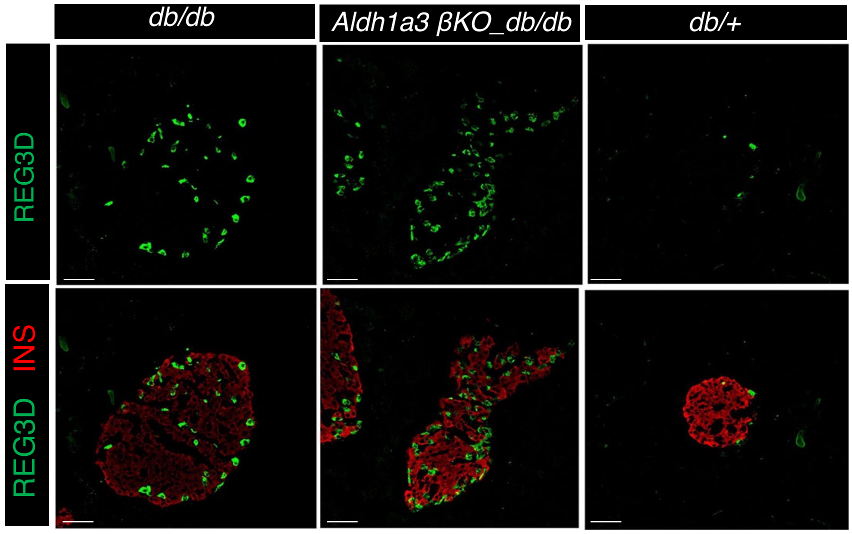

Supplementary Fig. 6 (A) Schematic diagram of RNA-Seq analysis. (B) Hierarchical clustering analysis using the whole gene expression profiles of β-Aldh1a3 KO_*db/db, lean and db/db* mice and heatmap visualization of TOP 50 highly expressed genes in β-Aldh1a3 KO_*db/db* compared to *db/db* control (Top) or in *db/db* control compared to lean mice (Bottom). (C) GO terms only enriched in β-Aldh1a3 KO_*db/db* compared to lean and *db/db* mice. (D) Co-immunostaining of REG3D (green) and Insulin (Red) using *db/db* (left), β-Aldh1a3 KO_*db/db* (middle), or in *db/+* (right) pancreata. Representative immunofluorescence images of n=4 mice per group. Scale bars: 50 μM.

Supplementary Fig. 7

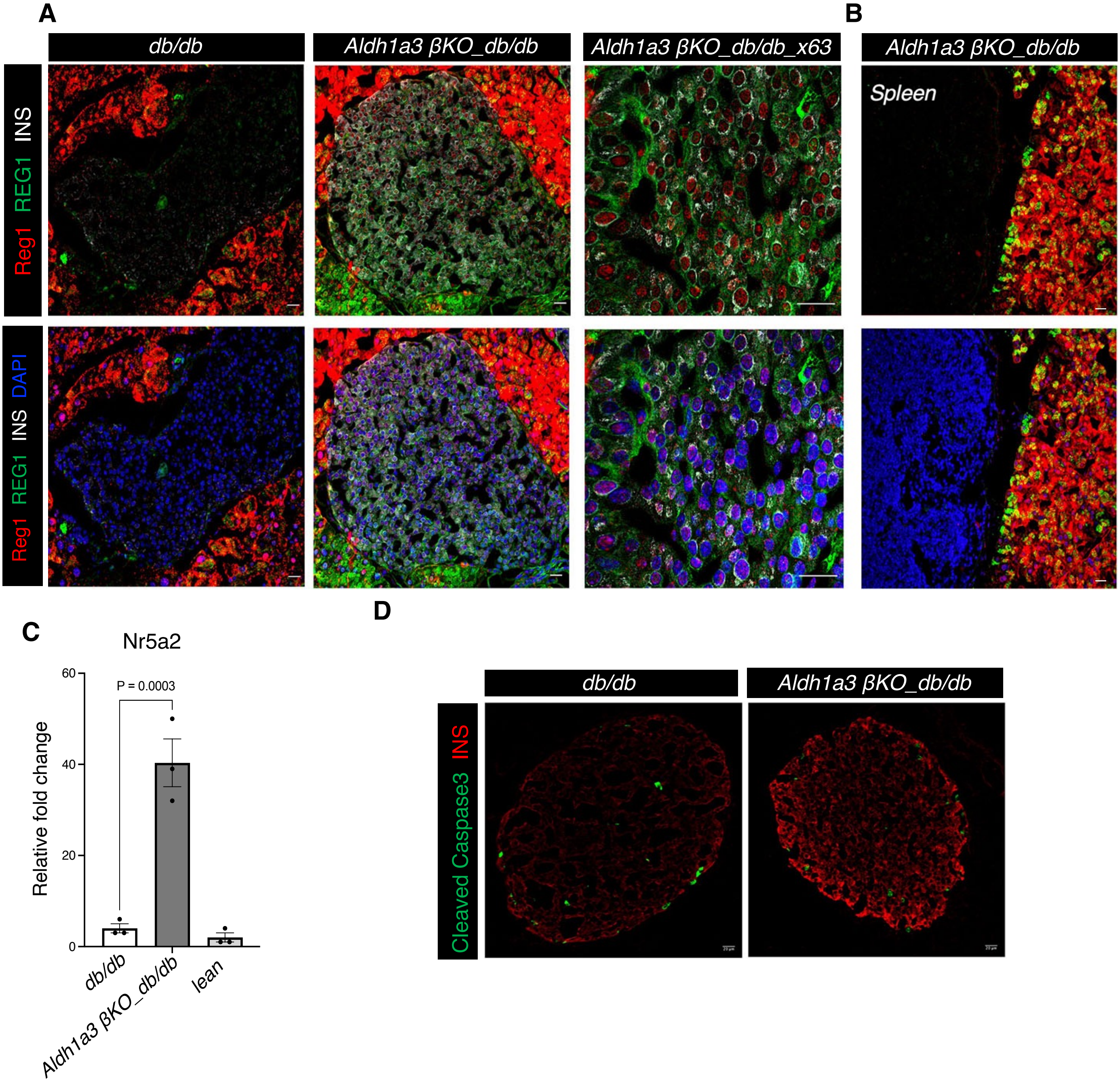

Supplementary Fig. 7 (A) *in situ Reg1* RNA hybridization and co-staining of REG1 and INS antibodies. Scale bars: 20 μM. (B) *in situ* RNA hybridization and co-staining of REG1 and INS antibodies in adjacent spleen sections. Representative immunofluorescence images of n=3 mice per group. Scale bars: 20 μM. (C) qPCR of Nr5a2 mRNA expression as indicated. Results expressed as fold changes relative to expression levels in lean control. Data are expressed as means ± SEM for n = 3 biologically independent samples per group. One-way ANOVA with multiple comparison test was used for statistical analysis. (D) Immunofluorescence of cleaved caspase3 as in (A). Representative immunofluorescence images of n=3 mice per group. Scale bars: 20 μM. Source data are provided as a Source Data file.

Supplementary Fig. 8

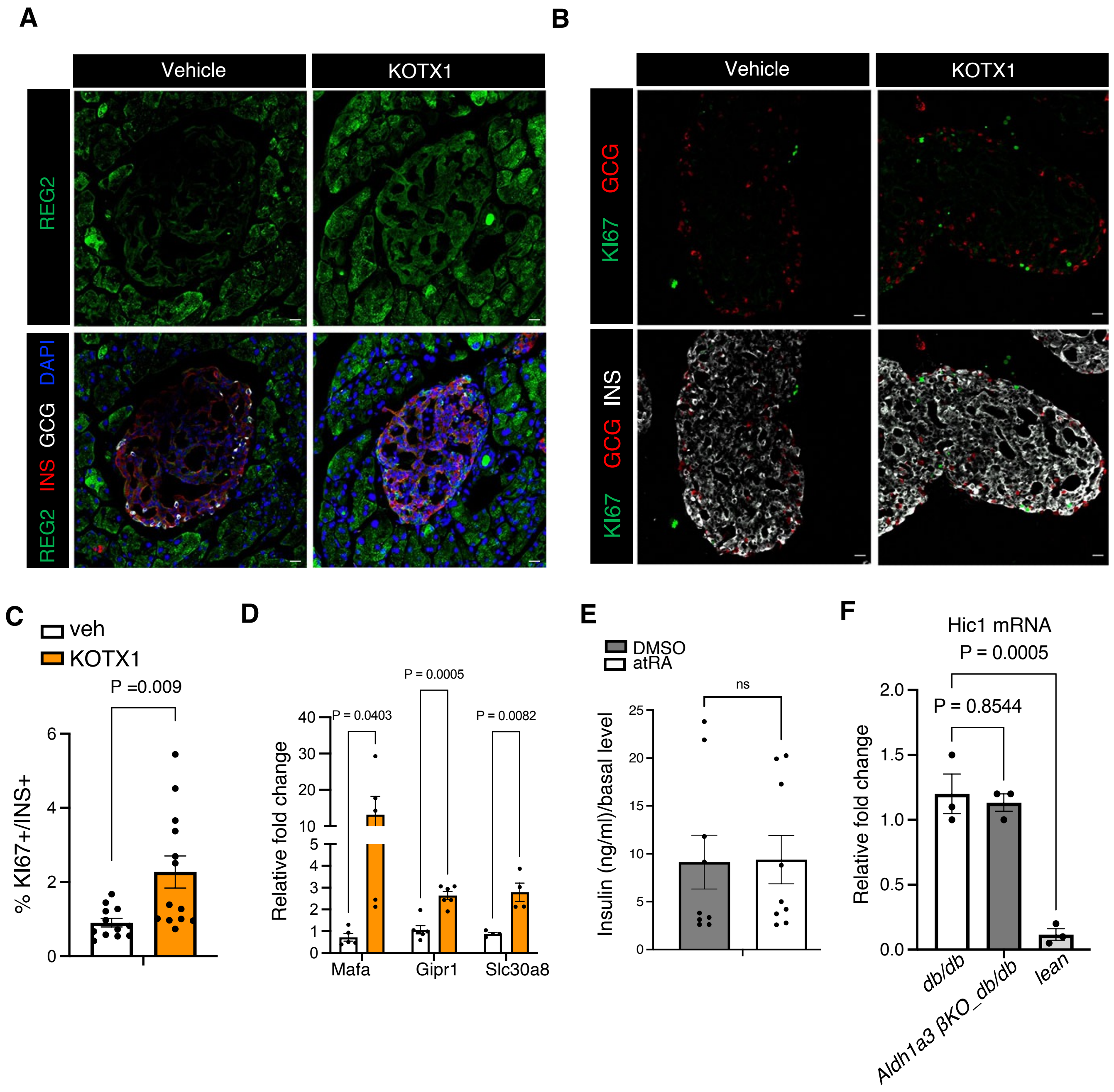

Supplementary Fig. 8 (A-B) Co-immunostaining of REG2 (A) or Ki67 (B), INS (Red), GCG or DAPI in KOTX1-treated *db/db* mice. Representative immunofluorescence images of n=5 mice per group. Scale bars: 20 μM. (C) Ratio of Ki67-positive cells in INS+ β-cell in KOTX1-treated *db/db* mice. Data are expressed as means ± SEM. N= 12 or 13 islets from 3 biologically independent samples per group. (D) qPCR analyses of selected genes using islets from KOTX- or vehicle-treated db/db mice. Results expressed as fold changes relative to expression levels in vehicle-control. Data are expressed as means ± SEM for n= minimum 4 biologically independent samples per group. Un-paired t-test with multiple comparison test was used for statistical analysis. (E) Glucose-induced insulin secretion in islets from control mice after treatment with KOTX1 or atRA. Data are expressed as means ± SEM. N=9 biologically independent samples per group. Two-tailed paired t-test was used for statistical analysis. (F) qPCR of Hic1 as indicated. Data are expressed as means ± SEM for n = 3 biologically independent samples per group. One-way ANOVA with multiple comparison test was used for statistical analysis. Source data are provided as a Source Data file.
