# Supplementary material for: Genetic and pharmacologic inhibition of ALDH1A3 as a treatment of β-cell failure
Source: Nat Commun. 2023 Feb 2;14:558. doi: 10.1038/s41467-023-36315-4 (PMC9895451; doi:10.1038/s41467-023-36315-4)
Supplement: Supplementary file 2 — Description of Additional Supplementary Files [file 41467_2023_36315_MOESM2_ESM.pdf]

Description of Additional Supplementary Files

Supplementary Data 1: DE gene list in multiple comparisons
