# Supplementary material for: Genetic and pharmacologic inhibition of ALDH1A3 as a treatment of β-cell failure
Source: Nat Commun. 2023 Feb 2;14:558. doi: 10.1038/s41467-023-36315-4 (PMC9895451; doi:10.1038/s41467-023-36315-4)
Supplement: Supplementary file 4 — Reporting Summary [file 41467_2023_36315_MOESM4_ESM.pdf]

nature portfolio

Corresponding author(s): Jinsook Son

Last updated by author(s): 2023_1_9

Reporting Summary

Nature Portfolio wishes to improve the reproducibility of the work that we publish. This form provides structure for consistency and transparency in reporting. For further information on Nature Portfolio policies, see our Editorial Policies and the Editorial Policy Checklist.

Statistics

For all statistical analyses, confirm that the following items are present in the figure legend, table legend, main text, or Methods section.

n/a	Confirmed	
☐	☒	The exact sample size (*n*) for each experimental group/condition, given as a discrete number and unit of measurement
☐	☒	A statement on whether measurements were taken from distinct samples or whether the same sample was measured repeatedly
☐	☒	The statistical test(s) used AND whether they are one- or two-sided<br>*Only common tests should be described solely by name; describe more complex techniques in the Methods section.*
☐	☒	A description of all covariates tested
☒	☐	A description of any assumptions or corrections, such as tests of normality and adjustment for multiple comparisons
☐	☒	A full description of the statistical parameters including central tendency (e.g. means) or other basic estimates (e.g. regression coefficient) AND variation (e.g. standard deviation) or associated estimates of uncertainty (e.g. confidence intervals)
☐	☒	For null hypothesis testing, the test statistic (e.g. *F*, *t*, *r*) with confidence intervals, effect sizes, degrees of freedom and *P* value noted<br>*Give P values as exact values whenever suitable.*
☒	☐	For Bayesian analysis, information on the choice of priors and Markov chain Monte Carlo settings
☒	☐	For hierarchical and complex designs, identification of the appropriate level for tests and full reporting of outcomes
☒	☐	Estimates of effect sizes (e.g. Cohen's *d*, Pearson's *r*), indicating how they were calculated

Our web collection on statistics for biologists contains articles on many of the points above.

Software and code

Policy information about availability of computer code

Data collection	NovaSeq 6000<br>BD Influx 1.2.0.108<br>Zeiss LSM 710 confocal laser scanning microscope
Data analysis	kallisto (version 0.44.0)<br>DESeq2 (version4.2)<br>Prism 9<br>fiji 2.0.0-rc-69/1.52p<br>Fcs express 7 (7.14.0020)<br>Zen 2.3

For manuscripts utilizing custom algorithms or software that are central to the research but not yet described in published literature, software must be made available to editors and reviewers. We strongly encourage code deposition in a community repository (e.g. GitHub). See the Nature Portfolio guidelines for submitting code & software for further information.

Data

Policy information about availability of data

All manuscripts must include a data availability statement. This statement should provide the following information, where applicable:

- Accession codes, unique identifiers, or web links for publicly available datasets
- A description of any restrictions on data availability
- For clinical datasets or third party data, please ensure that the statement adheres to our policy

The authors declare that all data supporting the findings of this study are available within the paper and/or the Supplementary Information/Source data file. RNA-Seq data have been deposited at the Gene Expression Omnibus (GEO) under accession number GSE218047. https://www.ncbi.nlm.nih.gov/geo/query/acc.cgi?acc=GSE211447

Human research participants

Policy information about studies involving human research participants and Sex and Gender in Research.

Reporting on sex and gender	*Use the terms sex (biological attribute) and gender (shaped by social and cultural circumstances) carefully in order to avoid confusing both terms. Indicate if findings apply to only one sex or gender; describe whether sex and gender were considered in study design whether sex and/or gender was determined based on self-reporting or assigned and methods used. Provide in the source data disaggregated sex and gender data where this information has been collected, and consent has been obtained for sharing of individual-level data; provide overall numbers in this Reporting Summary. Please state if this information has not been collected. Report sex- and gender-based analyses where performed, justify reasons for lack of sex- and gender-based analysis.*
Population characteristics	*Describe the covariate-relevant population characteristics of the human research participants (e.g. age, genotypic information, past and current diagnosis and treatment categories). If you filled out the behavioural & social sciences study design questions and have nothing to add here, write "See above."*
Recruitment	*Describe how participants were recruited. Outline any potential self-selection bias or other biases that may be present and how these are likely to impact results.*
Ethics oversight	*Identify the organization(s) that approved the study protocol.*

Note that full information on the approval of the study protocol must also be provided in the manuscript.

Field-specific reporting

Please select the one below that is the best fit for your research. If you are not sure, read the appropriate sections before making your selection.

[x] Life sciences [ ] Behavioural & social sciences [ ] Ecological, evolutionary & environmental sciences

For a reference copy of the document with all sections, see nature.com/documents/nr-reporting-summary-flat.pdf

Life sciences study design

All studies must disclose on these points even when the disclosure is negative.

Sample size	No sample size calculation was performed, and sample size was based on previous experience and common standards in similar field for calculating statistical significance. A minimum of three independent experiments were carried out. Sample numbers are indicated in the figure legends. Multiple tests and analyses were performed as described in the manuscript to ensure the samples are representative and results are conclusive.
Data exclusions	No data were excluded from the analysis.
Replication	The number of replicates for each specific experiment is indicated throughout the manuscript text, figure legends and methods.
Randomization	Age- and sex-matched mice were used in experiments to control the covariates.
Blinding	Investigators were blinded to group allocation during analysis of staining and performing physiological experiments.

Reporting for specific materials, systems and methods

We require information from authors about some types of materials, experimental systems and methods used in many studies. Here, indicate whether each material, system or method listed is relevant to your study. If you are not sure if a list item applies to your research, read the appropriate section before selecting a response.

Materials & experimental systems

n/a	Involved in the study	
☐	☒	Antibodies
☐	☒	Eukaryotic cell lines
☒	☐	Palaeontology and archaeology
☐	☒	Animals and other organisms
☒	☐	Clinical data
☒	☐	Dual use research of concern

Methods

n/a	Involved in the study	
☒	☐	ChIP-seq
☐	☒	Flow cytometry
☒	☐	MRI-based neuroimaging

Antibodies

Antibodies used	We used primary antibodies to INSULIN (A056401-2; Dako; 1:1000), GLUCAGON (G2654; clone K79bB10; Sigma-Aldrich; 1:1000), PDX1 (ab47308; Abcam; 1:100), E-cadherin (61018; BD Biosciences; 1:100), REG1 (AF1657; R&D systems; 1:100), REG2 (AF2035; R&D systems; 1:100), REG3d (MAB5678; Clone # 518818: R&D systems; 1:100), NR5a2 (PPH2325; R&D systems; 1:100), KI67 (GTX16667; Clone # SP6; Genetex; 1:100), MAFA (IHC-00352; Bethyl Laboratories; 1:100), Cleaved Caspase3 (9661; Cell Signaling; 1:100), and ALDH1A3 (NBP2-15339; Novus Biologicals; 1:100).
Validation	All antibodies we used were validated in the manufacture's product page and also validated in our previous work (Talchai et al., 2012, Cinti et al., 2016, Kim-muller et al., 2016).

Eukaryotic cell lines

Policy information about cell lines and Sex and Gender in Research

Cell line source(s)	A375 (ATCC CRL-1619)
Authentication	Cell lines were purchased from the ATCC (ATCC product documentation: https://www.atcc.org/products/crl-1619), but not independently authenticate them.
Mycoplasma contamination	All cell lines tested negative for mycoplasma.
Commonly misidentified lines (See ICLAC register)	None

Animals and other research organisms

Policy information about studies involving animals; ARRIVE guidelines recommended for reporting animal research, and Sex and Gender in Research

Laboratory animals	db/db (Lepr_db/db) mice and DIO mice (The Jackson Laboratory) were used for KOTX1 inhibitor studies.<br>Aldh1a3 CKO_db/db and Aldh1a3_CreERT knock in mice were generated and used in this study.<br>All mice were allowed ad libitum access to food and water and were maintained in specific pathogen-free conditions in a 22°C temperature-controlled room with a 12 h light-12 h dark cycle. Humidity maintained in the range of 40-70%.<br>All experiments were performed in 16- to 20-week-old male and female mice, unless specified otherwise in the figure legend.
Wild animals	No wild animals were used in the study.
Reporting on sex	We do not observe the sex differences for Aldh1a3 CKO_db/db and Aldh1a3_CreERT knock-in mice.<br><br>We only used male of db/db (Lepr_db/db) mice and DIO mice (The Jackson Laboratory) for KOTX1 inhibitor studies.
Field-collected samples	No field collected samples were used in the study.
Ethics oversight	All animal experiments were in accordance with NIH guidelines for Animal Care and Use, approved and overseen by Columbia University Institutional Animal Care and Use Committee (IACUC) under the study protocol AC-AABG6551.

Note that full information on the approval of the study protocol must also be provided in the manuscript.

Flow Cytometry

Plots

Confirm that:

- [x] The axis labels state the marker and fluorochrome used (e.g. CD4-FITC).
- [x] The axis scales are clearly visible. Include numbers along axes only for bottom left plot of group (a 'group' is an analysis of identical markers).
- [x] All plots are contour plots with outliers or pseudocolor plots.
- [x] A numerical value for number of cells or percentage (with statistics) is provided.

Methodology

Sample preparation	the animal was euthanized in a CO 2 chamber followed by cervical dislocation. The common bile duct was clamped with a hemostat near the liver, and 3 mL of 1 mg/mL cold Collagenase P (Sigma 11249002001) solution was injected into the hepatopancreatic ampulla to inflate the pancreas 49. The excised pancreas was incubated at 37 °C with shaking for 16 min. Medium 199 was added to a final volume of 50 mL, and the mixture centrifuged at 1100 rpm for 2 min at 4 °C. The pellet was resuspended with 10 mL of histopaque, and 10 mL of Medium 199 was layered on top of the histopaque followed by centrifugation at 2700 rpm for 20 min at 4 °C. Islets at the interface of the histopaque and Medium 199 were collected and washed twice with Medium 199 containing 10% FBS (Sigma-Aldrich F2442). Islets were handpicked into RPMI 1640 medium (ThermoFisher 11150-067) containing 15% FBS (Sigma-Aldrich F2442) for further analysis.
Instrument	BD Influx
Software	FCS express 7
Cell population abundance	Cell population abundance was determined via analysis with FCS express7. Purity of sorted cell populations were checked by qPCR for ALDH1A3, Insulin and Glucagon.
Gating strategy	The DEAB (pan-ALDH inhibitor)-treated cells were used as the negative control to set the gate. The same gate is applied to all samples in the same experiment. Gating strategy is presented in the supplementary figure.

- [x] Tick this box to confirm that a figure exemplifying the gating strategy is provided in the Supplementary Information.
